# Assessment of Genetically Modified Soybean in Relation to Natural Variation in the Soybean Seed Metabolome

**DOI:** 10.1038/srep03082

**Published:** 2013-10-30

**Authors:** Joseph D. Clarke, Danny C. Alexander, Dennis P. Ward, John A. Ryals, Matthew W. Mitchell, Jacob E. Wulff, Lining Guo

**Affiliations:** 1 Syngenta Biotechnology, Inc., 3504 Cornwallis Road, Research Triangle Park, NC 27709, USA; 2 Metabolon Inc., 617 Davis Drive, Suite 400, Durham, NC 27713, USA; 3 These authors contributed equally to this work.

## Abstract

Genetically modified (GM) crops currently constitute a significant and growing part of agriculture. An important aspect of GM crop adoption is to demonstrate safety and equivalence with respect to conventional crops. Untargeted metabolomics has the ability to profile diverse classes of metabolites and thus could be an adjunct for GM crop substantial equivalence assessment. To account for environmental effects and introgression of GM traits into diverse genetic backgrounds, we propose that the assessment for GM crop metabolic composition should be understood within the context of the natural variation for the crop. Using a non-targeted metabolomics platform, we profiled 169 metabolites and established their dynamic ranges from the seeds of 49 conventional soybean lines representing the current commercial genetic diversity. We further demonstrated that the metabolome of a GM line had no significant deviation from natural variation within the soybean metabolome, with the exception of changes in the targeted engineered pathway.

GM crops were first introduced to the market in 1994. Their adoption, especially in North and South America, has been steadily accelerating, now accounting for the majority of acreage in several major crops, including corn, soybean, cotton, and canola^1^,^2^. Transgenic herbicide tolerance and insect resistance have become the most widely applied trait types, providing the benefits of higher yield, lower input costs, and improved environmental profiles^2^,^3^,^4^. From the outset the most important aspect of GM crop adoption, and often the most controversial, has been whether they are as safe as conventional crops with respect to human and animal food consumption, as well as in their environmental impact. Extensive compositional and performance testing has always been required in order to gain regulatory approval for commercial release, and to date this concept has proven successful; despite more than a decade of widespread use of GM crops, and their extensive penetration into the food and feed chains, no case of deleterious effect on humans or animals has been found^2^. Of course, continued success requires case-by-case evidence that any new GM product meets similar or more refined standards.

The most common testing approach has been to compare the GM line to its nearest isogenic version, that is, the same line lacking only the transgenic insertion. Experience has shown that for traits in widespread use today effects of the transgene on the plant tend to be small, with the exception of parameters relating to the intended engineered trait. Indeed, testing over multiple years at multiple sites has shown that environmental effects on general metabolic variation within a particular line can be much greater than the variation due to the transgene itself 5,6,7,8. In addition, genetic variability within the commercial germplasm bearing an engineered trait can be quite diverse. In practice, conventional commercial crop lines have been individually optimized for performance in a wide variety of locations and environments, and a single GM line bearing a herbicide- or insect-resistance trait would not be economically viable outside the optimal agronomic region for that genetic background. As a result, the GM trait must be introgressed into a wide range of conventional backgrounds, and thus it is important to consider the variation of transgenic crops within the context of the entire range of germplasm expression.

A broad variety of methods have been used in the assessment of safety and equivalence for GM crops, and regulatory agencies rightly demand that the most advanced and accurate available technologies be applied. The most common approaches in the first decade of GM regulation involved extensive compositional and performance analysis for such characteristics as (in the case of animal feed) digestibility, gross levels of total protein, starch, fiber, fat, *etc.*, as well as targeted analysis of specific amino acids, fatty acids, secondary metabolites, and known toxins and anti-nutritive compounds^9^,^10^,^11^. As analytical technologies advanced it was suggested to expand the repertoire of analysis to include non-targeted “profiling” techniques^12^, with the reasoning that such analysis might give insight into unpredictable pleiotropic effects and potentially deleterious consequences of transgene expression^7^. In one example of the later case, negative alterations in expression might be related to the site of gene insertion, which in most cases is at a random site in the genome. Soon these profiling methods were being applied, including proteomics^13^, NMR-based metabolite fingerprinting^5^,^14^, HPLC or GC/MS metabolite analysis^5^,^8^, and global gene expression profiling^7^.

Because the trend for GM plant analysis has been toward more advanced and informative technologies, we have applied non-targeted global metabolomic analysis to the assessment of GM crops. Metabolomics, which is the global analysis of small molecule metabolites, is proving a powerful and sensitive technology for revealing perturbations in plant metabolic composition. We analyzed seeds from 49 conventional soybean lines and profiled 169 metabolites covering diverse biochemical pathways and classes. The relative levels of the metabolites across these lines provided a representation of the dynamic ranges of the natural soybean seed metabolome. As an example, we further analyzed a soybean GM line and found its metabolome resided well within the natural variation with the exception of the engineered pathway.

## Results

**Figure 1:**
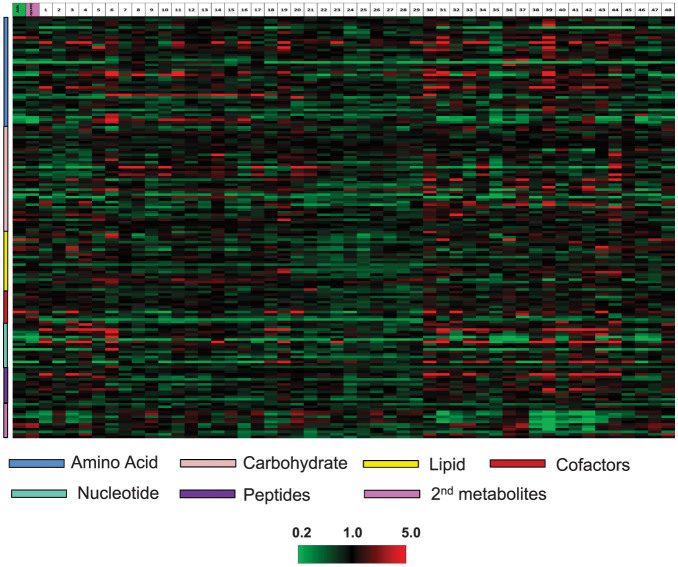
Metabolomic profiles and hierarchial clustering of 169 metabolites across the 49 soy conventional lines and one GM line. The mean values for 8 biological replications per line were shown. Red and green indicate high and low levels, respectively, respectively, relative to the median value for all samples (median = 1.0).

The metabolomics platform used in this analysis consists of three independent methods: ultrahigh performance liquid chromatography/tandem mass spectrometry (UHLC/MS/MS^2^) optimized for basic species, UHLC/MS/MS^2^ optimized for acidic species, and gas chromatography/mass spectrometry (GC/MS). The metabolites are identified by comparison of the ion features in the experimental samples to a reference library of chemical standard entries that include retention time, molecular weight (m/z), preferred adducts, and in-source fragments, as well as their associated MS/MS^2^ spectra. We analyzed seeds in eight biological replications from 49 conventional soybean accessions (Supplemental Table 1), which well represented the genetic diversity found in current commercial lines, and identified a total of 169 compounds of known structures, covering 51 biochemical pathways and compound types (Supplemental Figure 1). Interestingly, it was found that individual metabolites exhibited a considerable range of variation among the 49 conventional soybean lines (Figure 1). For each metabolite, we defined the natural dynamic range as the lines with the highest and lowest mean values. For individual metabolites the ratios of mean values for the line exhibiting the highest level to that having the lowest level ranged from 1.4-fold (stachyose) to >200-fold (allantoin) (Supplemental Table 2). This suggested that a portion of the soybean metabolome was tightly regulated across these lines, while the levels of many other metabolites could be dramatically altered by the combination of genetic and environmental impacts. Collectively, these data gave a representation of the natural variation range of the commercial soybean seed metabolome.

To test the effects of a genetically engineered trait on the soybean metabolome, we analyzed a soybean GM line resistant to the herbicide Mesotrione, and assessed the results in the context of metabolomic variation within a wide range of soybean germplasm. Mesotrione is a triketone herbicide which was developed based on the structure of a natural phytotoxin from *Callistemon* *citrinus*, the California bottlebrush plant, and acts as a competitive inhibitor of hydroxyphenylpyruvate dioxygenase (HPPD)^15^. Inhibition of HPPD leads to disruption of the carotenoid pathway, which is important in the production of photosynthetic electron transport compounds and anti-oxidant molecules in the tocopherol family. Blockage of this pathway allows runaway production of reactive oxygen species (ROS), which rapidly leads to light-induced chlorophyll damage and plant death. The GM trait was constructed in the soybean cultivar “Jack” by over-expressing a mutated oat HPPD gene, which conferred an ability to tolerate levels of mesotrione which are normally efficacious for control of dicot weeds.

We are not aware of established standards to judge the importance of potential metabolic perturbations which might result from transgene introduction, especially in the case of a large number of outcome variables. We thus propose different statistical methods to show what types of boundaries or comparisons might be considered, the goal being to develop hypotheses about the biological importance of any observed differences in transgenic lines relative to conventional germplasm. While it is not possible to establish “equivalence” by such methods, perturbations outside the normal ranges of conventional germplasm would certainly be a cause for concern and signal a need for further scrutiny.

**Figure 2:**
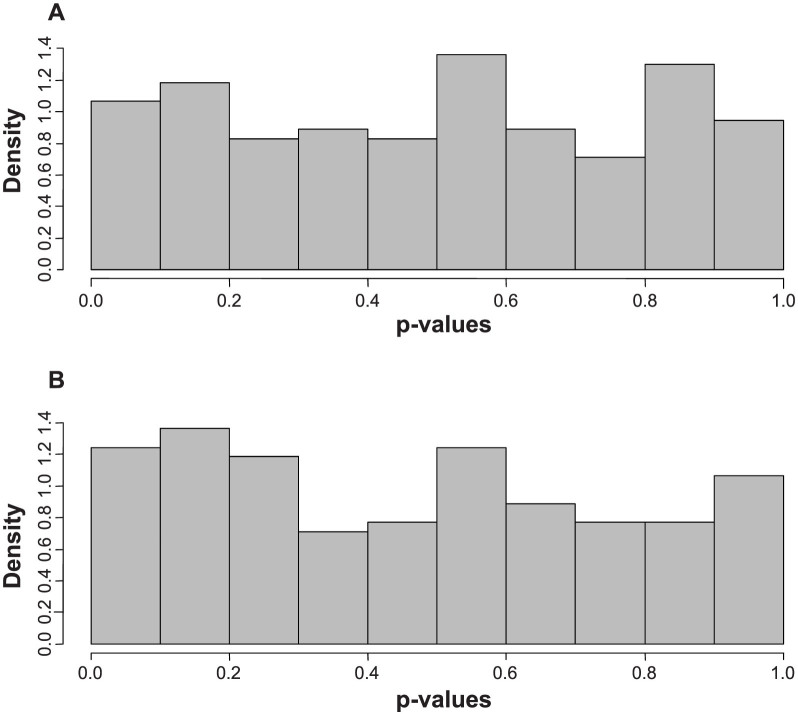
Histograms of p-value distributions for Jack (A) or the GM line (B) compared to 48 conventional (WT) soybean lines.

**Figure 3:**
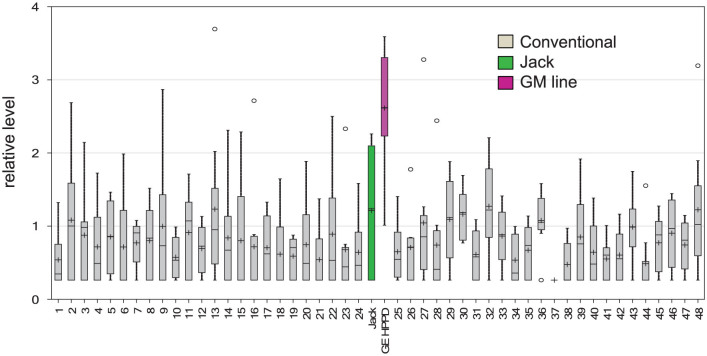
Box plot display the levels of delta-tocopherol of the GM line (pink) to its non-transgenic conventional parent Jack (green), and 48 other conventional soybean accessions (gray). The box represents the middle 50% of the distribution, and upper and lower “whiskers” represent the entire spread of the data. The hyphen refers to the line mean (n = 8) and circles represent outliers. The y-axis references the median scaled relative value.

In one approach we sought to establish if compound expression in the GM line differed from the various conventional lines, also termed “wild type” (WT) lines. The analysis used all of the conventional lines except for “Jack” as the baseline. Significance testing and prediction intervals were computed (Supplemental Table 3) comparing “Jack” *vs.* the other conventional WT lines, and then comparing the transgenic HPPD line (GM) *vs.* the WT lines (excluding “Jack”). A random effects model was fitted to the conventional lines with “LINE” treated as a random effect. The method of moments was used to estimate each variance component. Then prediction intervals for the average of a new line (with 8 seeds) were computed (first to compare the GM value, the second for “Jack”). The prediction intervals can be inverted to compute a p-value, which is equivalent to using the t-test with the pooled estimates of the variances (estimated from the 48 conventional lines). The histogram of the p-values for “Jack” vs. WT (Figure 2A) showed a uniform distribution, which was consistent with the case when there are no differences. By comparison, the GM p-value distribution (Figure 2B) shows a few potential metabolites that are different, in particular, the lowest two p-values were achieved by *delta*-tocopherol (Figure 3), and gamma-tocopherol, both products of the engineered pathway. A few other compounds showed higher but still relatively low p-values, *e.g.* AMP and phytate (*myo*-inositol-hexakisphosphate), which might flag them as compounds of interest. However, it is not possible to know if these result from some effect of the engineered gene, or if they represent false discoveries, several of which might be expected when performing 169 tests. We note that only *delta*-tocopherol meets the stricter multiple testing criterion of q < 0.1 for false discovery rate.

A minor drawback of the random effects model is that it can yield inaccurate estimates of confidence intervals and p-values for compounds which have significant missing data points, *e.g.* >50% nulls (see Supplemental Table 4). These compounds typically are represented by very small peaks, and can fall below the detectable limit in many of the lines tested. For instance, compounds such as N-carbamoylaspartate, in which all values were null for the GM line (as well as for 14 other lines), the low variance in the imputed data for the GM group would tend to underestimate p-values. However, because none of these approached real significance in the overall analysis, we feel this methodological shortcoming is not problematic for the question posed here.

**Figure 4:**
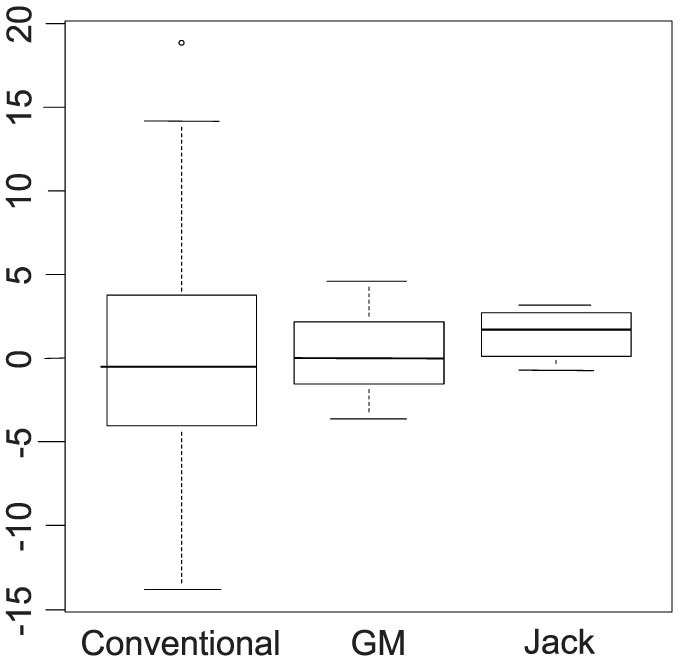
Predicted Values of the First Principal Component. The baseline was determined using the average value for each line (assuming the line is the experimental unit) for all the WT lines except “Jack”. The data were log-transformed, then each metabolite was centered and scaled by subtracting the mean of the transformed values and then dividing by the standard deviation of the transformed values. The first component accounted for approximately 27% of the total variance. The 95% Prediction Interval was (−9.17, 9.17), with means for GM and Jack equal to 0.35 and 1.44, respectively.

To avoid the issues related to multiple testing, we fitted a Principal Component Analysis (PCA) to the log-transformed data for the 48 lines, then the values for GM and “Jack” were predicted from this PCA. For simplicity, only the first component was used, but the results could be generalized to higher dimensions. The distributions of the predicted values for the first component are shown in the box plot diagrams in Figure 4. Next, the 95% prediction interval was computed for the mean of a new line (8 seeds), the result being (−9.17, 9.17). Both “Jack” (1.44) and the GM line (0.35) fell well within the prediction interval.

These analyses suggest that with the exception of the intended engineered trait, the overall metabolomic expression of the GM herbicide tolerant line could not readily be distinguished from conventional germplasm. Box plots for all biochemicals, comparing the GM line to the conventional line pool and to the parental line “Jack” and arranged by biochemical pathway group, are shown in Supplemental Figure 1.

## Discussion

Genes which encode genetically modified (GM) traits in crop plants for improved agronomic properties, such as tolerance to herbicides or resistance to insect attack, must be shown not to adversely affect the performance of the crop, nor to introduce unsafe alterations in food or feed products produced from these plants. Methods used to demonstrate the safety of GM crops have improved and expanded over the approximately 18 years since their introduction, with a trend toward inclusion of more global “-omics” technologies to gain a broad picture of plant biochemistry and physiology. Furthermore, the utility of any new method requires that it bring additional understanding of any potentially negative impacts that the engineered trait may have on the overall biochemical makeup or physiological performance of the target plant. Metabolomics, which is the global analysis of small molecule metabolites, is proving a powerful and sensitive technology for revealing perturbations, whether environmental or genetic, in plant metabolic compositions.

**Figure 5:**
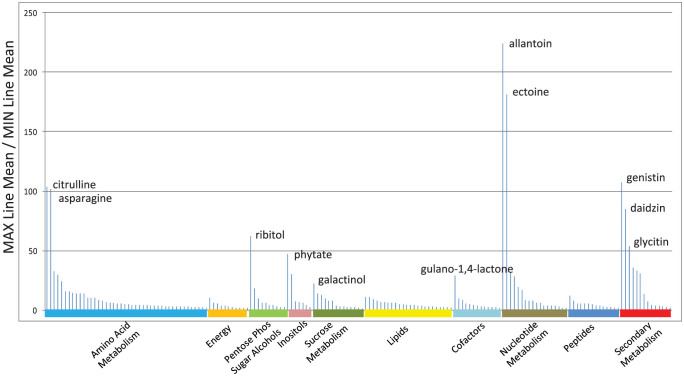
Distribution of metabolite variations between the means of the highest and lowest expressing lines by biochemical pathways and classes. The fold of differences between means of the highest and lowest expressing lines can be found in Supplemental Table 2.

We propose that comparing a GM line to the range of performance in a crop's native germplasm pool by metabolomic analysis could be a useful and proper standard as part of a safety and equivalence assessment program. It should be emphasized that the goal would be to uncover metabolic perturbations that should be further investigated in the context of performance and safety; of course, the absence of perturbations would not be sufficient to guarantee safety, but would only be part of the wider evaluation, including safety and environmental impacts of agronomic practices associated with the trait's application, such as herbicide safety, resistance, *etc.* We have now applied metabolomics to the question of equivalence assessment in the context of a genetically engineered crop plant, specifically to the effects of a herbicide tolerance gene in soybean. The metabolomic dataset here encompassed all the major pathways, including 44 compounds of the amino acid class, 42 carbohydrates, 24 lipids, 13 compounds in the cofactor/electron carrier class, 18 nucleotide derivatives, 14 peptides, and 14 compounds from secondary metabolism. The wide distribution of the metabolites measured, and the monitoring of multiple metabolites in key pathways such as glycolysis, the TCA cycle, nitrogen utilization, amino acid synthesis and catabolism, lipid oxidation, anti-oxidant utilization, and secondary metabolite production allowed many points of observation for any potential negative effects on metabolism caused by a transgene. It is interesting to consider the overall line-to-line metabolomic variation in commercial germplasm, and how a significant pleiotropic perturbation might express itself. More than half of the compounds showed <10-fold variation between the highest and lowest expressing lines (Supplemental Table 2), while some compounds showed a wide range of genotypic variation. As shown in Figure 5, energy metabolites, free fatty acids, and most amino acid metabolites were least variable, as might be expected in a metabolically quiescent tissue like seed, and negative effects on these pathways should be relatively easy to detect in a metabolomic screen. Compounds reflecting nutrient storage, environmental stress, and secondary metabolism were the most variable. For instance, citrulline, asparagine, and allantoin are all involved in nitrogen utilization and storage, while phytate is the storage sink for phosphate. These may vary strongly either from genetic factors, or because of nutrient availability during development. The compatible solutes ribitol, galactinol, and ectoine, as well as gulano-1,4-lactone (a precursor of ascorbate), are generated during seed desiccation, and may reflect environmental variation during seed maturation and desiccation. Accumulation of isoflavonoids in seeds (*e.g.* genistin, diadzin, glycitin) are also known to be subject to both genetic and environmental control^16^,^17^,^18^. Good pathway coverage, and a knowledge of expected natural metabolomic diversity demonstrates the usefulness of the method for monitoring metabolic consequences of an engineered trait, and the present case provides strong evidence that the seed's physiology was not disturbed in a significant manner by the presence of the transgene. As more complex traits are developed to impact more general crop performance, such as yield, drought tolerance, nitrogen utilization, *etc.*, and which will likely involve engineered regulatory genes, it will be increasingly important to understand a trait's impacts on a broad range of biochemical pathways. Metabolomic analysis is ideally suited for such a task.

## Methods

### Materials

Seeds from 49 conventional soybean lines (Supplemental Table 1) were obtained from the USDA National Soybean Research Center at University of Illinois and Syngenta Biotechnology, Inc. Seed for the herbicide-tolerant GM line (event SYHT06W) was obtained from Syngenta. Eight individual mature seeds for each soybean line were subjected to metabolomic analysis.

### Metabolomic profiling

The metabolomic platforms consisted of three independent platforms: ultrahigh performance liquid chromatography/tandem mass spectrometry (UHLC/MS/MS^2^) optimized for basic species, UHLC/MS/MS^2^ optimized for acidic species, and gas chromatography/mass spectrometry (GC/MS). The detailed descriptions of these platforms, including instrument, data acquisition and processing, and compound identification and quantitation, were published previously^19^,^20^. Essentially, the samples were extracted and split into three equal aliquots for analysis on the three platforms. For the two LC platforms, chromatographic separation followed by full scan mass spectra was carried out to record retention time, molecular weight (m/z) and MS/MS^2^ of all detectable ions presented in the samples. For the GC platform, the samples were derivatized using bistrimethyl-silyl-triflouroacetamide (BSTFA). The retention time and molecular weight (m/z) for all detectable ions were measured. The metabolites were identified by comparison of the ion features in the experimental samples to a reference library of chemical standard entries that included retention time, molecular weight (m/z), preferred adducts, and in-source fragments as well as their associated MS/MS^2^ spectra.

### Data imputation and statistical analysis

Integrated peak ion counts were used to compare relative levels of a compound in each sample. For statistical analysis, the missing values for a given metabolite were imputed with the observed minimum detected value based on the assumption that they were below the limits of instrument detection sensitivity. Supplemental Table 4 shows the frequency of present/missing data; values represent the percentage of the eight samples for each line in which the compound was detected. Statistical analysis of the data was performed using “R” (http://cran.r-project.org/). For graphical display in heat maps data for each biochemical were scaled to the median observed value for that compound, then missing values were imputed as described above. For box plot displays the data were further transformed to log2 values. Multiple comparisons were accounted for using the q-value method of Storey and Tibshirani^21^ (Supplemental Table 3).

## Author Contributions

D.W., J.C. and L.G. proposed the original concept and study design. D.W., D.A., J.R. and L.G. performed data analysis and interpretation. M.M. and J.W. did the statistical analysis. All authors contributed to the writing of the manuscript.

## Supplementary Material

Supplementary Information supplemental material
