# Supplementary material for: Assessment of Genetically Modified Soybean in Relation to Natural Variation in the Soybean Seed Metabolome
Source: Sci Rep. 2013 Oct 30;3:3082. doi: 10.1038/srep03082 (PMC3812653; doi:10.1038/srep03082)

Supplemental Material

Assessment of Genetically Modified Soybean in Relation to Natural Variation in the Soybean Seed Metabolome

Joseph D. Clarke, Danny C. Alexander, Dennis P. Ward, John A. Ryals, Matthew W. Mitchell, Jacob E. Wulff, Lining Guo

Supplemental Table 1: Soybean lines used in this study.

Line Designation	Trait Type	Maturity Group	Source
03RM893031	Conventional	III	Syngenta
05RM926125	Conventional	V	Syngenta
93B87/M816626	Conventional	IV	Syngenta
97021-A00-60059 /97177-N00-22972	Conventional	II	Syngenta
ApeX SY-308001	Conventional	III	USDA
BI 13404BB2/A2722 PLTTBR	Conventional	III	Syngenta
BPR99402 (WTBBl)/99022-A01-16277-01 (PLTTBL)	Conventional	III	Syngenta
Bradley 01S-4028	Conventional	00	USDA
Chippewa 64 06U-1144	Conventional	00	USDA
Colquitt 64 06U-1144	Conventional	VII	USDA
Curtis 03S-5067	Conventional	VI	USDA
DST2343/PXB23Y02	Conventional	III	Syngenta
Dunbar 08U-3047	Conventional	III	USDA
Dwight 05U-1266	Conventional	III	USDA
Egyptian 07U-5745	Conventional	IV	USDA
Erie 06U-4319	Conventional	V	USDA
Gasoy 17 04S-1339	Conventional	VII	USDA
Glenwood 08U-1049	Conventional	0	USDA
Govan 04S-1342	Conventional	VI	USDA
Hagwood 04S-1344	Conventional	VII	USDA
Hill 05S-119	Conventional	V	USDA
Hutcheson 05S-109	Conventional	V	USDA
Jack	Conventional	III	Syngenta
Lloyd 03S-5050	Conventional	VI	USDA
NE0800088	Conventional	V	Syngenta
NE0800094	Conventional	V	Syngenta
NE0800097	Conventional	V	Syngenta
NE1900 08U-1120	Conventional	I	USDA
P93B86/M815869	Conventional	IV	Syngenta
Pana 09U-3563	Conventional	IV	USDA
Ransom 04S-1351	Conventional	IV	USDA
S23-T5	Conventional	II	Syngenta
S23-T5 5.2SA S12-C2/S25-H5 SCN	Conventional	II	Syngenta
S25-J5	Conventional	II	Syngenta
S32-Z3/SJ734826	Conventional	IV	Syngenta
S33-A8	Conventional	III	Syngenta
S42-H1	Conventional	IV	Syngenta
Semmes 04S-1352	Conventional	VII	USDA
SG801122200/96601-B99-17498	Conventional	IV	Syngenta
SJ0800021	Conventional	IV	Syngenta
SJ833009	Conventional	IV	Syngenta
SYHT06W(Jack)	GM	III	Syngenta
TN 4-86 08U-1654	Conventional	IV	USDA
W115926/93B15	Conventional	III	Syngenta
Williams-82	Conventional	III	Syngenta

Winchester 07U-5570	Conventional	000	USDA
Wright 04S-1358	Conventional	VI	USDA
WW115926 (WLtBBl)/98211-A01-36153 (PGTIB)	Conventional	III	Syngenta
WW221162	Conventional	III	Syngenta
XB33R02/X006	Conventional	III	Syngenta

Supplemental Table 2: The ratios between the lines with the highest level and the lowest level for each metabolite.

BIOCHEMICAL	Super Pathway	Sub Pathway	RATIO
allantoin	Nucleotide	Purine metabolism	224.0
ectoine	Nucleotide	Pyrimidine metabolism	181.2
genistin	Secondary metabolism	Flavonoids	107.4
citrulline	Amino acid	Glutamate family (alpha-ketoglutarate derived)	103.4
asparagine	Amino acid	Aspartate family (OAA derived)	101.8
daidzin	Secondary metabolism	Flavonoids	85.1
ribitol	Carbohydrate	Amino sugar and nucleotide sugar	62.0
glycitin	Secondary metabolism	Flavonoids	53.7
genistein	Secondary metabolism	Flavonoids	35.6
trans-4-hydroxyproline	Amino acid	Glutamate family (alpha-ketoglutarate derived)	32.5
adenosine	Nucleotide	Purine metabolism	32.2
glycitein	Secondary metabolism	Flavonoids	31.4
daidzein	Secondary metabolism	Flavonoids	31.0
chiro-inositol	Carbohydrate	Inositol metabolism	29.9
gulono-1,4-lactone	Cofactors, Prosthetic Groups, Electron Carriers	Ascorbate metabolism	29.3
putrescine	Amino acid	Amines and polyamines	28.7
guanosine-2',3'-cyclic monophosphate	Nucleotide	Purine metabolism	28.4
myo-inositol hexakisphosphate	Carbohydrate	Inositol metabolism	26.2
4-hydroxycinnamate	Amino acid	Aromatic amino acid metabolism (PEP derived)	23.9
galactinol	Carbohydrate	Sucrose, glucose, fructose metabolism	22.2
adenosine-2',3'-cyclic monophosphate	Nucleotide	Purine metabolism	19.6
arabitol	Carbohydrate	Amino sugar and nucleotide sugar	18.3
allantoic acid	Nucleotide	Purine metabolism	16.9
beta-alanine	Amino acid	Aspartate family (OAA derived)	15.6
pyroglutamine*	Amino acid	Glutamate family (alpha-ketoglutarate derived)	15.6
arginine	Amino acid	Glutamate family (alpha-ketoglutarate derived)	14.6
agmatine	Amino acid	Amines and polyamines	14.1
N-carbamoylaspartate	Amino acid	Aspartate family (OAA derived)	13.8
1-kestose	Carbohydrate	Sucrose, glucose, fructose metabolism	13.7
naringenin	Secondary metabolism	Flavonoids	13.3
galactitol (dulcitol)	Carbohydrate	Sucrose, glucose, fructose metabolism	12.7
gamma-glutamylhistidine	Peptide	gamma-glutamyl	12.2
glycerophosphorylcholine (GPC)	Lipids	Phospholipids	11.2
2-hydroxypalmitate	Lipids	Free fatty acid	11.1
histidine	Amino acid	Glutamate family (alpha-ketoglutarate derived)	10.5
N6-acetyllysine	Amino acid	Aspartate family (OAA derived)	10.5
malate	Carbohydrate	TCA cycle	10.4
homoserine	Amino acid	Aspartate family (OAA derived)	10.2
xylitol	Carbohydrate	Amino sugar and nucleotide sugar	9.5
mannitol	Carbohydrate	Sucrose, glucose, fructose metabolism	9.5
linolenate [alpha or gamma; (18:3n3 or 6)]	Lipids	Free fatty acid	9.4
spermidine	Amino acid	Amines and polyamines	9.4
2-aminoadipate	Amino acid	Aspartate family (OAA derived)	8.8
pyridoxate	Cofactors, Prosthetic Groups, Electron Carriers	Vitamin B metabolism (B6 or B12)	8.6
adenosine 3'-monophosphate (3'-AMP)	Nucleotide	Purine metabolism	8.2
2-hydroxyadipate	Lipids	Free fatty acid	8.2
2'-deoxyadenosine	Nucleotide	Purine metabolism	8.1
adenosine 5'-monophosphate (AMP)	Nucleotide	Purine metabolism	7.9
fructose	Carbohydrate	Sucrose, glucose, fructose metabolism	7.9
gamma-glutamylvaline	Peptide	gamma-glutamyl	7.9
sorbitol	Carbohydrate	Sucrose, glucose, fructose metabolism	7.8
2-isopropylmalate	Amino acid	Branched Chain Amino Acids (pyruvate derived)	7.7
myo-inositol	Carbohydrate	Inositol metabolism	7.6
syringic acid	Secondary metabolism	Flavonoids	7.1
linoleate (18:2n6)	Lipids	Free fatty acid	7.0
2-hydroxystearate	Lipids	Free fatty acid	6.8
methylsuccinate	Amino acid	Branched Chain Amino Acids (pyruvate derived)	6.8
pinitol	Carbohydrate	Inositol metabolism	6.6
1,3-dihydroxyacetone	Carbohydrate	Glycolysis	6.4
tryptophan	Amino acid	Aromatic amino acid metabolism (PEP derived)	6.3
campesterol	Lipids	Sterols	6.2
erythritol	Carbohydrate	Amino sugar and nucleotide sugar	6.2
xanthosine	Nucleotide	Purine metabolism	6.1
cytidine-3'-monophosphate (3'-CMP)	Nucleotide	Pyrimidine metabolism	6.0
scyllo-inositol	Carbohydrate	Inositol metabolism	6.0
2-hydroxyglutarate	Lipids	Free fatty acid	6.0
serine	Amino acid	Serine family (phosphoglycerate derived)	6.0
1-palmitoylglycerophosphoinositol*	Lipids	Phospholipids	6.0
gluconate	Carbohydrate	Amino sugar and nucleotide sugar	6.0
gamma-glutamylisoleucine*	Peptide	gamma-glutamyl	5.7
gamma-glutamylmethionine	Peptide	gamma-glutamyl	5.7
threonate	Cofactors, Prosthetic Groups, Electron Carriers	Ascorbate metabolism	5.7
4-acetamidobutanoate	Amino acid	Glutamate family (alpha-ketoglutarate derived)	5.6
glucose	Carbohydrate	Glycolysis	5.5
pipecolate	Amino acid	Aspartate family (OAA derived)	5.4
pyroglutamylglutamine	Peptide	Dipeptide	5.4
alanylleucine	Peptide	Dipeptide	5.3
1-stearoylglycerophosphoinositol	Lipids	Lysolipids	5.2
gamma-glutamyltryptophan	Peptide	gamma-glutamyl	5.1
choline phosphate	Lipids	Choline metabolism	5.1
gamma-aminobutyrate (GABA)	Amino acid	Glutamate family (alpha-ketoglutarate derived)	4.9
aspartate	Amino acid	Aspartate family (OAA derived)	4.9
delta-tocopherol	Cofactors, Prosthetic Groups, Electron Carriers	Tocopherol metabolism	4.9
nicotinamide riboside*	Cofactors, Prosthetic Groups, Electron Carriers	Nicotinate and nicotinamide metabolism	4.6
azelate (nonanedioate)	Lipids	Free fatty acid	4.5
vanillate	Secondary metabolism	Benzenoids	4.5
glycerol 2-phosphate	Lipids	Phospholipids	4.4
erythronate*	Carbohydrate	Amino sugar and nucleotide sugar	4.3
methionine sulfoxide	Amino acid	Aspartate family (OAA derived)	4.3
threitol	Carbohydrate	Amino sugar and nucleotide sugar	4.3
3-methyl-2-oxovalerate	Amino acid	Branched Chain Amino Acids (pyruvate derived)	4.2
glycine	Amino acid	Serine family (phosphoglycerate derived)	4.2
stearamide	Lipids	Fatty acid amide	4.2
inositol 2-phosphate (I2P)	Carbohydrate	Inositol metabolism	4.1
leucine	Amino acid	Branched Chain Amino Acids (pyruvate derived)	4.1
glutamate	Amino acid	Glutamate family (alpha-ketoglutarate derived)	4.0
alpha-glutamylglutamate	Peptide	Dipeptide	4.0
uridine-2',3'-cyclic monophosphate	Nucleotide	Pyrimidine metabolism	3.9
glutamine	Amino acid	Glutamate family (alpha-ketoglutarate derived)	3.9
dimethylarginine (SDMA + ADMA)	Amino acid	Glutamate family (alpha-ketoglutarate derived)	3.9
N-acetylornithine	Amino acid	Glutamate family (alpha-ketoglutarate derived)	3.9
gamma-glutamylglutamate	Peptide	gamma-glutamyl	3.9
cytidine	Nucleotide	Pyrimidine metabolism	3.8
4-methyl-2-oxopentanoate	Amino acid	Branched Chain Amino Acids (pyruvate derived)	3.8
glucarate 1,4-lactone	Carbohydrate	Sucrose, glucose, fructose metabolism	3.8
pantothenate	Cofactors, Prosthetic Groups, Electron Carriers	CoA metabolism	3.8
dihydrokaempferol	Secondary metabolism	Flavonoids	3.8
oleate (18:1n9)	Lipids	Free fatty acid	3.8
guanine	Nucleotide	Purine metabolism	3.7
succinate	Carbohydrate	TCA cycle	3.6
cytosine	Nucleotide	Pyrimidine metabolism	3.6
13-HODE + 9-HODE	Lipids	Oxylipins	3.6
guanosine	Nucleotide	Purine metabolism	3.4
cysteine	Amino acid	Serine family (phosphoglycerate derived)	3.4
glycerol 3-phosphate (G3P)	Lipids	Phospholipids	3.3
glycerate	Carbohydrate	Glycolysis	3.3
gamma-tocopherol	Cofactors, Prosthetic Groups, Electron Carriers	Tocopherol metabolism	3.3
alanine	Amino acid	Aspartate family (OAA derived)	3.3
naringenin-7-O-glucoside	Secondary metabolism	Flavonoids	3.3
beta-sitosterol	Lipids	Sterols	3.3
malonate (propanedioate)	Lipids	Free fatty acid	3.3
galactose	Carbohydrate	Sucrose, glucose, fructose metabolism	3.2
valine	Amino acid	Branched Chain Amino Acids (pyruvate derived)	3.2
methylphosphate	Cofactors, Prosthetic Groups, Electron Carriers	Oxidative phosphorylation	3.2
isoleucine	Amino acid	Branched Chain Amino Acids (OAA derived)	3.1
lysine	Amino acid	Aspartate family (OAA derived)	3.1
alpha-ketoglutarate	Carbohydrate	TCA cycle	3.0
glycyltyrosine	Peptide	Dipeptide	3.0
citramalate	Carbohydrate	C5 branched dibasic acid metabolism	3.0
threonine	Amino acid	Aspartate family (OAA derived)	2.9
arabonate	Carbohydrate	Amino sugar and nucleotide sugar	2.9
tyrosine	Amino acid	Aromatic amino acid metabolism (PEP derived)	2.9
glucarate (saccharate)	Cofactors, Prosthetic Groups, Electron Carriers	Ascorbate metabolism	2.8
stigmasterol	Secondary metabolism	Terpenoids	2.8
verbascose	Carbohydrate	Sucrose, glucose, fructose metabolism	2.8
proline	Amino acid	Glutamate family (alpha-ketoglutarate derived)	2.8
phenylalanine	Amino acid	Aromatic amino acid metabolism (PEP derived)	2.8
nicotinamide ribonucleotide (NMN)	Cofactors, Prosthetic Groups, Electron Carriers	Nicotinate and nicotinamide metabolism	2.7
methionine	Amino acid	Aspartate family (OAA derived)	2.7
phosphate	Cofactors, Prosthetic Groups, Electron Carriers	Oxidative phosphorylation	2.7
inositol 1-phosphate (I1P)	Carbohydrate	Inositol metabolism	2.7
glycerol	Lipids	Glycerolipids	2.6
fumarate	Carbohydrate	TCA cycle	2.6
3-deoxyoctulosonate	Carbohydrate	Sucrose, glucose, fructose metabolism	2.6
ribose	Carbohydrate	Amino sugar and nucleotide sugar	2.5
xylonate	Carbohydrate	Amino sugar and nucleotide sugar	2.5
5-oxoproline	Amino acid	Glutathione metabolism	2.5
1-palmitoylglycerol (1-monopalmitin)	Lipids	Glycerolipids	2.5
2-palmitoylglycerol (2-monopalmitin)	Lipids	Glycerolipids	2.5
gamma-glutamyltyrosine	Peptide	gamma-glutamyl	2.4
galactarate (mucic acid)	Secondary metabolism	Fatty acid and sugar derivatives	2.4
valylglutamate	Peptide	Dipeptide	2.4
ethanolamine	Lipids	Choline metabolism	2.4
raffinose	Carbohydrate	Sucrose, glucose, fructose metabolism	2.3
trigonelline (N'-methylnicotinate)	Cofactors, Prosthetic Groups, Electron Carriers	Nicotinate and nicotinamide metabolism	2.3
p-hydroxybenzaldehyde	Secondary metabolism	Benzenoids	2.2
isocitrate	Carbohydrate	TCA cycle	2.1
nicotinate ribonucleoside*	Cofactors, Prosthetic Groups, Electron Carriers	Nicotinate and nicotinamide metabolism	2.0
caproate (6:0)	Lipids	Free fatty acid	2.0
adenine	Nucleotide	Purine metabolism	2.0
pyruvate	Carbohydrate	Glycolysis	2.0
gamma-glutamylglutamine	Peptide	gamma-glutamyl	2.0
4-guanidinobutanoate	Amino acid	Serine family (phosphoglycerate derived)	1.9
uridine	Nucleotide	Pyrimidine metabolism	1.9
cis-aconitate	Carbohydrate	TCA cycle	1.8
sucrose	Carbohydrate	Sucrose, glucose, fructose metabolism	1.7
gamma-glutamylphenylalanine	Peptide	gamma-glutamyl	1.7
citrate	Carbohydrate	TCA cycle	1.6
stachyose	Carbohydrate	Sucrose, glucose, fructose metabolism	1.4

Supplemental Table 3: Predition Intervals and Significance Testing. Compounds are sorted by ascending GM *vs* . WT p-values. The tests are described in the RESULTS and METHODS sections.

Var1 = estimated LINE variance component
Var2 = estimated residual variance
Prop1 = proportion of total variance from LINE (= var1/(var1 + var2))
Prop2 = proportion of total variance from the residual (= var2/(var1 + var2))
PI_LOW = the lower limit of a 95% prediction for the mean of a new line
PI_UPP = the upper limit if a 95% prediction for the mean of a new line
MEAN_GM = the mean value of the GM line (mean of the log-transformed)
MEAN_JACK = the mean value of the JACK line (mean of the log-transformed)
pval_GM = p-value of GM vs. WT (excluding Jack)
pval_JACK = p-value of Jack vs. WT (excluding Jack)
qval_GM or JACK = corresponding q-value

BIOCHEMICAL	VAR1	VAR2	PROP 1	PROP 2	PI_LOW	PI_UPP	MEAN GM	MEAN JACK	pval GM	*qval GM*	pval JACK	*qval JACK*
delta-tocopherol	0.0113	0.6126	0.0181	0.9819	-1.2287	-0.0237	0.5620	-0.2769	0.0002	*0.0390*	0.2493	*0.9309*
gamma-tocopherol	0.0373	0.2105	0.1506	0.8494	-1.4262	-0.4008	-0.1341	-0.4095	0.0037	*0.2903*	0.0539	*0.8387*
adenosine 5'-monophosphate (AMP)	0.1271	0.2302	0.3558	0.6442	-0.3098	1.2953	-0.5952	-0.1373	0.0090	*0.3694*	0.1210	*0.8596*
myo-inositol-hexakisphosphate	0.8081	1.0052	0.4457	0.5543	-1.5257	2.4025	-2.0781	-0.5122	0.0131	*0.3694*	0.3352	*0.9509*
alpha-ketoglutarate	0.0674	0.3233	0.1724	0.8276	-0.7084	0.6262	-0.8958	-0.5402	0.0132	*0.3694*	0.1391	*0.8596*
glutamate	0.0480	0.1144	0.2955	0.7045	-0.5326	0.4819	-0.6524	-0.6478	0.0165	*0.3694*	0.0173	*0.6656*
arginine	0.2296	0.5266	0.3037	0.6963	-1.1528	1.0568	-1.4021	-1.1659	0.0174	*0.3694*	0.0475	*0.8387*
pyruvate	0.0170	0.0636	0.2105	0.7895	-0.6032	0.0383	0.0994	0.0937	0.0206	*0.3694*	0.0225	*0.6656*
spermidine	0.3128	0.5460	0.3642	0.6358	-0.9097	1.5996	-1.1417	-0.3234	0.0212	*0.3694*	0.2894	*0.9405*
ectoine	1.9843	0.4464	0.8164	0.1836	-3.7516	2.0548	-4.1912	-3.9732	0.0249	*0.3694*	0.0355	*0.7493*
homoserine	0.1690	0.5130	0.2478	0.7522	-1.4260	0.5366	-1.5686	-0.9506	0.0257	*0.3694*	0.3050	*0.9509*
2-aminoadipate	0.1215	0.2496	0.3273	0.6727	-1.0693	0.5192	0.5692	1.1272	0.0377	*0.4971*	0.0009	*0.1492*
citramalate	0.0297	0.1763	0.1441	0.8559	-0.6304	0.2940	-0.6403	-0.0730	0.0455	*0.5532*	0.6806	*0.9666*
methylphosphate	0.0255	0.2974	0.0790	0.9210	-0.5743	0.4435	-0.5633	-0.2554	0.0550	*0.5798*	0.4564	*0.9509*
2-hydroxyadipate	0.1868	0.1667	0.5284	0.4716	-1.2988	0.5537	0.5336	0.8356	0.0550	*0.5798*	0.0117	*0.6656*
putrescine	0.5366	0.4665	0.5349	0.4651	-1.7342	1.4012	-1.6526	-1.1914	0.0626	*0.6192*	0.1948	*0.8665*
stearamide	0.0744	0.5543	0.1183	0.8817	-1.0340	0.5068	-0.9740	-0.8941	0.0699	*0.6502*	0.1064	*0.8596*
gamma-glutamylglutamine	0.0157	0.0654	0.1932	0.8068	-0.4485	0.1794	0.1483	0.1254	0.0763	*0.6637*	0.1025	*0.8596*
lysine	0.0318	0.0934	0.2539	0.7461	-0.4753	0.3723	-0.4271	-0.1896	0.0811	*0.6637*	0.5154	*0.9509*
2-hydroxyglutarate	0.0921	0.1329	0.4092	0.5908	-1.0039	0.3363	0.2399	-0.3927	0.0916	*0.6637*	0.8603	*0.9814*
adenosine 3'-monophosphate (3'-AMP)	0.1644	0.3198	0.3395	0.6605	-0.9688	0.8689	-0.8285	-0.8730	0.0949	*0.6637*	0.0780	*0.8387*
syringic acid	0.2344	0.2883	0.4484	0.5516	-0.6043	1.5097	1.3262	0.9362	0.1031	*0.6637*	0.3621	*0.9509*
alpha-glutamylglutamate	0.0527	0.1083	0.3272	0.6728	-0.5707	0.4753	0.3828	0.0441	0.1044	*0.6637*	0.7255	*0.9814*
malate	0.2255	0.1171	0.6583	0.3417	-1.2596	0.7325	0.5325	0.2695	0.1146	*0.6637*	0.2871	*0.9405*
nicotinamide-riboside*	0.1206	0.0924	0.5662	0.4338	-1.0434	0.4346	0.2709	0.3543	0.1241	*0.6637*	0.0794	*0.8387*
glycitein	0.3482	1.1299	0.2356	0.7644	-1.8917	0.9523	0.6172	0.7567	0.1308	*0.6637*	0.0893	*0.8596*
valine	0.0324	0.0697	0.3177	0.6823	-0.5560	0.2687	-0.4522	-0.3401	0.1390	*0.6637*	0.3428	*0.9509*
guanosine-2-3-cyclic-monophosphate	0.4040	0.6226	0.3935	0.6065	-2.1357	0.6862	-1.7748	-1.6656	0.1410	*0.6637*	0.1862	*0.8596*
linolenate [alpha or gamma] (18:3n3 or 6)	0.1791	0.5617	0.2418	0.7582	-1.4785	0.5514	0.2887	0.7161	0.1426	*0.6637*	0.0237	*0.6656*
dihydrokaempferol	0.2086	0.3745	0.3578	0.6422	-1.9291	0.1253	-1.6629	-1.4209	0.1428	*0.6637*	0.3147	*0.9509*
citrulline	0.8115	1.4057	0.3660	0.6340	-3.4919	0.5471	-2.9565	-2.5636	0.1460	*0.6637*	0.2826	*0.9405*
verbascose	0.0299	0.0745	0.2866	0.7134	-0.4804	0.3248	0.2174	-0.0540	0.1469	*0.6637*	0.9059	*0.9814*
adenosine-2-3-cyclic-monophosphate	0.3205	0.5092	0.3863	0.6137	-1.8529	0.6668	-1.5158	-1.4740	0.1473	*0.6637*	0.1661	*0.8596*
N-acetylornithine	0.0915	0.0715	0.5612	0.4388	-0.6707	0.6174	-0.4971	0.0145	0.1484	*0.6637*	0.8982	*0.9814*
proline	0.0373	0.0694	0.3494	0.6506	-0.6004	0.2712	0.1494	-0.0605	0.1539	*0.6637*	0.6331	*0.9666*
gamma-glutamylisoleucine*	0.1614	0.2643	0.3791	0.6209	-1.0994	0.6932	-0.8458	-0.7325	0.1558	*0.6637*	0.2407	*0.9247*
pyroglutamine*	0.5626	0.1633	0.7750	0.2250	-1.9911	1.1129	-1.5515	-1.2760	0.1559	*0.6637*	0.2835	*0.9405*
agmatine	0.2646	0.5577	0.3218	0.6782	-1.8528	0.4978	-1.5074	-1.5674	0.1621	*0.6637*	0.1344	*0.8596*
glycitin	0.3710	1.1434	0.2450	0.7550	-1.7255	1.1889	0.7365	0.7156	0.1719	*0.6637*	0.1808	*0.8596*
galactinol	0.5672	0.4017	0.5854	0.4146	-1.9341	1.2600	-1.4320	-0.6105	0.1744	*0.6637*	0.7321	*0.9814*
N6-acetyllysine	0.1968	0.7996	0.1975	0.8025	-2.0613	0.1531	-1.7131	-1.8574	0.1744	*0.6637*	0.1074	*0.8596*
alanylleucine	0.0827	0.1386	0.3736	0.6264	-0.7435	0.5419	-0.5360	-0.1706	0.1796	*0.6637*	0.8279	*0.9814*
pipecolate	0.1000	0.1241	0.4464	0.5536	-0.9145	0.4673	0.2433	0.1602	0.1805	*0.6637*	0.2695	*0.9405*
asparagine	0.4375	0.8885	0.3299	0.6701	-1.7349	1.2759	-1.2180	-0.8671	0.1929	*0.6668*	0.3985	*0.9509*
p-hydroxybenzaldehyde	0.0256	0.1049	0.1962	0.8038	-0.7982	0.0018	-0.6556	-0.6218	0.2018	*0.6668*	0.2666	*0.9405*
4-guanidinobutanoate	0.0296	0.0426	0.4097	0.5903	-0.4429	0.3164	0.1805	0.0630	0.2029	*0.6668*	0.5068	*0.9509*
guanine	0.0446	0.4492	0.0903	0.9097	-0.8859	0.4041	-0.6545	-0.9769	0.2034	*0.6668*	0.0262	*0.6656*
isocitrate	0.0147	0.0840	0.1493	0.8507	-0.2795	0.3663	-0.1623	0.0710	0.2062	*0.6668*	0.8642	*0.9814*
threonate	0.0812	0.1315	0.3817	0.6183	-0.8099	0.4600	-0.5755	-0.3373	0.2107	*0.6668*	0.6093	*0.9509*
threonine	0.0302	0.0929	0.2452	0.7548	-0.5437	0.2875	-0.3902	-0.1362	0.2108	*0.6668*	0.9689	*0.9875*
scyllo-inositol	0.1246	0.4133	0.2317	0.7683	-1.5863	0.1205	-1.2600	-1.2728	0.2202	*0.6817*	0.2093	*0.9072*
isoleucine	0.0459	0.0676	0.4043	0.5957	-0.6562	0.2913	-0.4708	-0.4969	0.2269	*0.6817*	0.1882	*0.8596*
glycerol 2-phosphate	0.1104	0.2921	0.2742	0.7258	-0.9234	0.6346	-0.6169	-0.6715	0.2284	*0.6817*	0.1799	*0.8596*
5-oxoproline	0.0375	0.0553	0.4042	0.5958	-0.5855	0.2713	0.0901	-0.1801	0.2515	*0.7278*	0.9144	*0.9843*
glycine	0.0442	0.0953	0.3169	0.6831	-0.6498	0.3131	-0.4402	-0.3329	0.2617	*0.7278*	0.4951	*0.9509*
erythritol	0.1090	0.2223	0.3290	0.6710	-1.0650	0.4386	-0.7373	-0.2339	0.2621	*0.7278*	0.8328	*0.9814*
pyroglutamylglutamine	0.1396	0.3542	0.2827	0.7173	-1.2724	0.4708	0.0907	-0.4270	0.2623	*0.7278*	0.9521	*0.9875*
gulono 1,4-lactone	0.6983	0.2925	0.7048	0.2952	-2.4114	1.0734	0.3028	0.9570	0.2676	*0.7297*	0.0667	*0.8387*
gamma-glutamylphenylalanine	0.0163	0.0352	0.3169	0.6831	-0.4126	0.1724	0.0408	-0.1271	0.2741	*0.7311*	0.9620	*0.9875*
glycerate	0.1059	0.2550	0.2934	0.7066	-0.7321	0.7768	-0.3846	-0.1802	0.2834	*0.7311*	0.5917	*0.9509*
adenine	0.0247	0.0536	0.3151	0.6849	-0.5911	0.1290	-0.0372	0.0137	0.2843	*0.7311*	0.1779	*0.8596*
gamma-glutamylvaline	0.1802	0.2540	0.4150	0.5850	-1.1085	0.7632	-0.6696	-0.4734	0.2908	*0.7311*	0.5211	*0.9509*
allantoin	1.0191	1.2501	0.4491	0.5509	-2.7265	1.6806	-1.6922	-1.3944	0.2912	*0.7311*	0.4303	*0.9509*
mannitol	0.2199	0.1009	0.6855	0.3145	-1.1606	0.7998	0.3331	0.0913	0.2973	*0.7348*	0.5797	*0.9509*
linoleate (18:2n6)	0.1356	0.3081	0.3056	0.6944	-1.2801	0.4162	0.0037	0.1969	0.3067	*0.7424*	0.1425	*0.8596*
gamma-glutamylglutamate	0.0423	0.0855	0.3311	0.6689	-0.5993	0.3366	-0.3685	-0.1455	0.3131	*0.7424*	0.9517	*0.9875*
pantothenate	0.0798	0.0348	0.6967	0.3033	-0.6773	0.5023	0.2078	0.1485	0.3189	*0.7424*	0.4248	*0.9509*
nicotinate ribonucleoside*	0.0333	0.0683	0.3276	0.6724	-0.7054	0.1257	-0.0832	0.0309	0.3222	*0.7424*	0.1272	*0.8596*
histidine	0.2581	0.3226	0.4445	0.5555	-1.2198	1.0010	-0.6597	-0.7476	0.3239	*0.7424*	0.2534	*0.9309*
leucine	0.0938	0.0903	0.5096	0.4904	-0.8548	0.4632	-0.5161	-0.3768	0.3331	*0.7527*	0.5833	*0.9509*
sorbitol	0.2192	0.2502	0.4670	0.5330	-1.7293	0.3053	-0.2319	0.2087	0.3473	*0.7736*	0.0750	*0.8387*
chiro-inositol	0.4790	0.1487	0.7631	0.2369	-1.7589	1.1087	0.3330	0.1925	0.3605	*0.7757*	0.4713	*0.9509*
threitol	0.1184	0.1592	0.4264	0.5736	-0.9965	0.5152	-0.5854	-0.0632	0.3635	*0.7757*	0.6389	*0.9666*
gamma-glutamyltryptophan	0.1589	0.1671	0.4874	0.5126	-1.0990	0.6246	0.1529	0.0323	0.3672	*0.7757*	0.5324	*0.9509*
stigmasterol	0.0425	0.6515	0.0612	0.9388	-0.9996	0.4315	0.0394	0.1612	0.3678	*0.7757*	0.2168	*0.9161*
gamma-glutamylhistidine	0.2681	0.4049	0.3983	0.6017	-1.3923	0.9025	-0.7540	-0.9460	0.3767	*0.7839*	0.2251	*0.9240*
ribose	0.0289	0.1082	0.2107	0.7893	-0.6918	0.1453	-0.0975	0.0294	0.4025	*0.8268*	0.1523	*0.8596*
allantoic acid	0.3525	0.6155	0.3642	0.6358	-2.2877	0.3764	-1.5074	-1.0896	0.4089	*0.8292*	0.8406	*0.9814*

BIOCHEMICAL	VAR1	VAR2	PROP 1	PROP 2	PI_LOW	PI_UPP	MEAN GM	MEAN JACK	pval GM	qval GM	pval JACK	qval JACK
serine	0.0626	0.1329	0.3202	0.6798	-0.8312	0.3129	-0.4925	-0.3042	0.4160	0.8318	0.8748	0.9814
cysteine	0.0569	0.2322	0.1968	0.8032	-0.7104	0.4812	-0.3516	-0.1522	0.4277	0.8318	0.8994	0.9814
2'-deoxyadenosine	0.0504	0.3487	0.1262	0.8738	-1.3065	-0.0605	-0.9308	-0.8410	0.4285	0.8318	0.6133	0.9509
N-carbamoylaspartate	0.4935	0.4074	0.5478	0.4522	-2.7293	0.2702	-1.8198	-1.5762	0.4325	0.8318	0.6441	0.9666
3-methyl-2-oxovalerate	0.0944	0.1689	0.3586	0.6414	-0.8243	0.5576	-0.3992	-0.0257	0.4428	0.8318	0.7553	0.9814
glycerophosphorylcholine (GPC)	0.3528	0.3411	0.5084	0.4916	-1.2574	1.2989	-0.4710	-0.3733	0.4428	0.8318	0.5381	0.9509
alanine	0.0744	0.0917	0.4479	0.5521	-0.8615	0.3296	-0.4902	-0.4696	0.4527	0.8318	0.4950	0.9509
azelate (nonanedioate)	0.0344	0.2807	0.1092	0.8908	-0.9970	0.0747	-0.6612	-0.7009	0.4564	0.8318	0.3726	0.9509
4-acetamidobutanoate	0.1176	0.3187	0.2696	0.7304	-1.1373	0.4756	-0.6199	-0.2597	0.4744	0.8318	0.8600	0.9814
adenosine	0.6618	0.3988	0.6240	0.3760	-1.4294	2.0000	-0.3278	-0.3499	0.4755	0.8318	0.4598	0.9509
ribitol	1.1439	0.4965	0.6973	0.3027	-3.1464	1.3179	-1.6876	-2.0374	0.4893	0.8318	0.3166	0.9509
xylitol	0.3197	0.3680	0.4648	0.5352	-1.6446	0.8137	-0.8277	-0.8568	0.5032	0.8318	0.4737	0.9509
glycyltyrosine	0.0217	0.3479	0.0587	0.9413	-0.6861	0.3520	-0.3411	-0.0568	0.5033	0.8318	0.6712	0.9666
fumarate	0.0309	0.1183	0.2070	0.7930	-0.3098	0.5590	0.2690	0.2370	0.5069	0.8318	0.6051	0.9509
galactitol (dulcitol)	0.1411	0.4637	0.2333	0.7667	-1.3012	0.5126	-0.0968	-0.3168	0.5126	0.8318	0.8644	0.9814
arabitol	0.0901	0.4097	0.1803	0.8197	-1.0636	0.4646	-0.0498	-0.1413	0.5140	0.8318	0.6789	0.9666
4-methyl-2-oxopentanoate	0.1064	0.1805	0.3707	0.6293	-0.8232	0.6366	-0.3282	-0.0276	0.5206	0.8318	0.8570	0.9814
gamma-glutamyltyrosine	0.0356	0.0455	0.4387	0.5613	-0.4380	0.3880	-0.1570	-0.1474	0.5232	0.8318	0.5537	0.9509
2-palmitoylglycerol (2-monopalmitin)	0.0000	0.2045	0.0000	1.0000	-0.8577	-0.2077	-0.4293	-0.8282	0.5250	0.8318	0.0738	0.8387
ethanolamine	0.0080	0.1284	0.0584	0.9416	-0.4599	0.1702	-0.0449	-0.0617	0.5262	0.8318	0.5980	0.9509
valinylglutamate	0.0231	0.1850	0.1108	0.8892	-0.5755	0.2983	-0.2767	-0.2849	0.5278	0.8318	0.5037	0.9509
galactose	0.0449	0.1279	0.2598	0.7402	-0.7742	0.2289	-0.1151	-0.1113	0.5304	0.8318	0.5208	0.9509
13-HODE-9-HODE	0.0546	0.0558	0.4946	0.5054	-0.8319	0.1772	-0.4856	-0.2754	0.5311	0.8318	0.8369	0.9814
cytosine	0.0479	0.2523	0.1597	0.8403	-0.6622	0.4839	0.0840	0.2912	0.5462	0.8470	0.1882	0.8596
campesterol	0.0169	0.7315	0.0225	0.9775	-1.1636	0.1742	-0.2988	-0.2756	0.5586	0.8546	0.5130	0.9509
choline phosphate	0.1216	0.2363	0.3397	0.6603	-0.2969	1.2833	0.2693	0.0215	0.5713	0.8546	0.2357	0.9247
phosphate	0.0387	0.0450	0.4622	0.5378	-0.5473	0.3082	-0.2406	-0.0466	0.5719	0.8546	0.7331	0.9814
uridine-2',3'-cyclic-monophosphate	0.0290	0.0603	0.3243	0.6757	-0.7535	0.0231	-0.4731	-0.4731	0.5787	0.8546	0.5787	0.9509
daidzin	1.1493	0.4463	0.7203	0.2797	-2.7646	1.6980	-1.1449	-0.7087	0.5840	0.8546	0.8750	0.9814
sucrose	0.0214	0.0125	0.6312	0.3688	-0.4402	0.1757	-0.0502	-0.0258	0.5947	0.8546	0.4902	0.9509
glucarate (saccharate)	0.0573	0.1161	0.3306	0.6694	-0.6601	0.4294	-0.2599	0.3882	0.5961	0.8546	0.0692	0.8387
4-hydroxycinnamate	0.5516	0.9488	0.3676	0.6324	-2.7497	0.5783	-0.6489	-0.8210	0.5999	0.8546	0.7504	0.9814
1-palmitoylglycerol (1-monopalmitin)	0.0435	0.0769	0.3611	0.6389	-0.6217	0.3148	-0.0313	0.0898	0.6024	0.8546	0.3015	0.9509
1-palmitoylglycerophosphoinositol*	0.2506	0.1549	0.6181	0.3819	-1.1487	0.9637	0.1749	0.0608	0.6129	0.8546	0.7716	0.9814
daidzein	0.6326	0.3700	0.6310	0.3690	-2.3790	0.9703	-1.1266	-0.6594	0.6144	0.8546	0.9571	0.9875
2-isopropylmalate	0.0372	0.3151	0.1056	0.8944	-0.9493	0.1756	-0.5280	-0.7869	0.6161	0.8546	0.1591	0.8596
vanillate	0.0606	0.1309	0.3165	0.6835	-0.2512	0.8765	0.4512	0.1039	0.6235	0.8546	0.4601	0.9509
arabonate	0.0705	0.1152	0.3797	0.6203	-0.8625	0.3223	-0.1281	0.1627	0.6319	0.8546	0.1483	0.8596
glutamine	0.0445	0.2337	0.1599	0.8401	-0.6398	0.4636	-0.2202	-0.1276	0.6322	0.8546	0.8860	0.9814
succinate	0.0629	0.1383	0.3127	0.6873	-0.6876	0.4636	-0.2418	-0.3880	0.6522	0.8585	0.3397	0.9509
cytidine	0.0561	0.2057	0.2143	0.7857	-0.7901	0.3727	-0.0782	-0.0516	0.6536	0.8585	0.5892	0.9509
dimethylarginine (ADMA + SDMA)	0.0816	0.1747	0.3184	0.6816	-0.7547	0.5528	0.0450	-0.1759	0.6555	0.8585	0.8187	0.9814
beta-alanine	0.1025	0.3911	0.2077	0.7923	-1.4533	0.1284	-0.8368	-0.6147	0.6595	0.8585	0.9038	0.9814
pyridoxate	0.2989	0.2227	0.5731	0.4269	-1.5845	0.7391	-0.1688	-0.2338	0.6622	0.8585	0.7450	0.9814
nicotinamide-ribonucleotide, (NMN)	0.0329	0.0754	0.3040	0.6960	-0.2887	0.5481	0.0417	-0.3433	0.6741	0.8668	0.0276	0.6656
trans-4-hydroxyproline	0.1349	0.4654	0.2247	0.7753	-1.5379	0.2484	-0.8292	-0.9880	0.6797	0.8670	0.4434	0.9509
naringenin-7-O-glucoside	0.0693	0.1226	0.3610	0.6390	-1.0786	0.1037	-0.6067	-0.6868	0.6868	0.8690	0.5009	0.9509
glycerol	0.0433	0.0918	0.3203	0.6797	-0.7284	0.2230	-0.1617	-0.2225	0.7019	0.8795	0.8989	0.9814
inositol-1-phosphate (I1P)	0.0590	0.0615	0.4893	0.5107	-0.5797	0.4698	0.0438	0.0080	0.7067	0.8795	0.8104	0.9814
caproate (6:0)	0.0258	0.0811	0.2415	0.7585	-0.3964	0.3745	-0.0783	-0.2011	0.7268	0.8795	0.3261	0.9509
1,3-dihydroxyacetone	0.1509	0.6187	0.1960	0.8040	-1.3057	0.6363	-0.5030	0.3180	0.7288	0.8795	0.1827	0.8596
3-deoxyoctulosonate	0.0206	0.6118	0.0325	0.9675	-1.0631	0.2033	-0.3209	-0.5637	0.7304	0.8795	0.6728	0.9666
2-hydroxypalmitate	0.1779	0.2012	0.4694	0.5306	-0.9636	0.8684	-0.2013	-0.2160	0.7373	0.8795	0.7132	0.9814
myo-inositol	0.2022	0.1369	0.5963	0.4037	-1.2620	0.6419	-0.4683	-0.2888	0.7395	0.8795	0.9643	0.9875
xylonate	0.0551	0.0781	0.4137	0.5863	-0.6765	0.3590	-0.2448	-0.0892	0.7396	0.8795	0.7883	0.9814
phenylalanine	0.0499	0.0521	0.4891	0.5109	-0.6625	0.3026	-0.1045	-0.2122	0.7544	0.8904	0.8937	0.9814
1-kestose	0.6078	0.2622	0.6986	0.3014	-2.1831	1.0706	-0.3128	-1.0996	0.7647	0.8959	0.5049	0.9509
methionine	0.0352	0.0357	0.4962	0.5038	-0.4397	0.3698	-0.0896	-0.1668	0.7870	0.9003	0.5154	0.9509
methylsuccinate	0.0463	0.2496	0.1565	0.8435	-1.1971	-0.0654	-0.5563	-0.7091	0.7910	0.9003	0.7831	0.9814
2-hydroxystearate	0.0997	0.3205	0.2373	0.7627	-0.9885	0.5315	-0.3251	-0.5588	0.7992	0.9003	0.3864	0.9509
tyrosine	0.0404	0.0707	0.3636	0.6364	-0.5560	0.3458	-0.1596	-0.0580	0.8087	0.9003	0.8347	0.9814
gamma-aminobutyrate (GABA)	0.1126	0.2305	0.3282	0.6718	-1.0840	0.4446	-0.2293	-0.2254	0.8129	0.9003	0.8050	0.9814
erythronate*	0.0769	0.2794	0.2159	0.7841	-1.0049	0.3547	-0.4003	-0.3652	0.8248	0.9003	0.9059	0.9814
methionine sulfoxide	0.0838	0.0973	0.4627	0.5373	-0.8677	0.3915	-0.1691	-0.1962	0.8266	0.9003	0.8942	0.9814
uridine	0.0172	0.0386	0.3080	0.6920	-0.5185	0.0845	-0.1846	-0.0346	0.8297	0.9003	0.2296	0.9240
stachyose	0.0060	0.0071	0.4583	0.5417	-0.2263	0.1121	-0.0752	-0.0305	0.8301	0.9003	0.7529	0.9814
oleate (18:1n9)	0.1163	0.1012	0.5347	0.4653	-1.1368	0.3227	-0.3337	-0.0938	0.8406	0.9003	0.3923	0.9509
beta-sitosterol	0.0617	0.3161	0.1633	0.8367	-0.8376	0.4558	-0.1264	-0.0473	0.8419	0.9003	0.6571	0.9666
cytidine 3'-monophosphate (3'-CMP)	0.0753	0.3395	0.1816	0.8184	-1.1411	0.2538	-0.3761	-0.4491	0.8464	0.9003	0.9875	0.9875
malonate-propanedioate-	0.0813	0.0485	0.6264	0.3736	-0.7494	0.4520	-0.2046	-0.1407	0.8523	0.9003	0.9788	0.9875
gamma-glutamylmethionine	0.1341	0.2443	0.3543	0.6457	-1.2023	0.4472	-0.3011	-0.6294	0.8528	0.9003	0.5419	0.9509
xanthosine	0.1514	0.1300	0.5382	0.4618	-0.9483	0.7164	-0.1926	0.1024	0.8539	0.9003	0.6002	0.9509
gluconate	0.1299	0.2092	0.3831	0.6169	-1.2741	0.3320	-0.5261	-0.2481	0.8910	0.9259	0.5791	0.9509
glycerol 3-phosphate (G3P)	0.0813	0.0566	0.5893	0.4107	-0.6153	0.5931	-0.0479	-0.2269	0.9032	0.9259	0.4760	0.9509
cis-aconitate	0.0217	0.0108	0.6676	0.3324	-0.3703	0.2464	-0.0806	0.0175	0.9038	0.9259	0.6070	0.9509
guanosine	0.0361	0.1794	0.1675	0.8325	-0.6528	0.3307	-0.1901	-0.0135	0.9058	0.9259	0.5491	0.9509
1-stearoylglycerophosphoinositol	0.1454	0.1499	0.4924	0.5076	-1.1838	0.4632	-0.3174	-0.1865	0.9171	0.9259	0.6732	0.9666
raffinose	0.0436	0.0370	0.5409	0.4591	-0.5476	0.3455	-0.1239	-0.1075	0.9185	0.9259	0.9769	0.9875
genistein	0.4687	0.3227	0.5922	0.4078	-2.1016	0.7987	-0.5922	-0.4244	0.9348	0.9259	0.7541	0.9814
aspartate	0.1174	0.2188	0.3492	0.6508	-0.9284	0.6183	-0.1860	0.0210	0.9361	0.9259	0.6490	0.9666
pinitol	0.1861	0.0983	0.6544	0.3456	-1.0849	0.7259	-0.1471	-0.1950	0.9430	0.9259	0.9726	0.9875
citrate	0.0104	0.0226	0.3160	0.6840	-0.3128	0.1555	-0.0867	0.0256	0.9451	0.9259	0.3749	0.9509
glucose	0.1781	0.1702	0.5113	0.4887	-1.0124	0.8027	-0.1347	0.1490	0.9474	0.9259	0.5763	0.9509
inositol 2-phosphate (I2P)	0.0524	0.4060	0.1144	0.8856	-1.0612	0.2446	-0.3878	-0.3394	0.9498	0.9259	0.8329	0.9814
glucarate-1-4-lactone	0.0773	0.1943	0.2845	0.7155	-0.8403	0.4551	-0.2068	0.3541	0.9648	0.9259	0.0962	0.8596
naringenin	0.1765	0.1451	0.5488	0.4512	-1.4976	0.2957	-0.5823	-0.6113	0.9668	0.9259	0.9817	0.9875
tryptophan	0.1601	0.1632	0.4951	0.5049	-1.1823	0.5446	-0.3341	-0.6372	0.9718	0.9259	0.4620	0.9509
fructose	0.2686	0.1897	0.5860	0.4140	-1.2165	0.9813	-0.0999	0.2357	0.9742	0.9259	0.5209	0.9509
galactarate (mucic acid)	0.0291	0.0566	0.3396	0.6604	-0.4052	0.3679	-0.0241	0.1196	0.9776	0.9259	0.4754	0.9509
trigonelline (N-methylnicotinate)	0.0417	0.0566	0.4242	0.5758	-0.4406	0.4575	0.0050	-0.1891	0.9876	0.9298	0.3807	0.9509
genistin	0.9323	0.3649	0.7187	0.2813	-2.5439	1.4762	-0.5331	-0.4759	0.9994	0.9353	0.9540	0.9875

Supplemental Table 4: Percentage of samples in which each metabolite was detected in the soybean lines.

BIOCHEMICAL	JACK	GM	L1	L2	L3	L4	L5	L6	L7	L8	L9	L10	L11	L12	L13	L14	L15	L16	L17	L18	L19	L20	L21	L22	L23	L24	L25	L26	L27	L28	L29	L30	L31	L32	L33	L34	L35	L36	L37	L38	L39	L40	L41	L42	L43	L44	L45	L46	L47	L48
1,3-dihydroxyacetone	100	100	100	62.5	75	87.5	75	50	100	100	87.5	87.5	87.5	62.5	62.5	87.5	50	87.5	87.5	87.5	100	100	87.5	62.5	75	75	87.5	87.5	87.5	50	87.5	100	75	100	100	62.5	100	87.5	100	100	75	100	100	75	87.5	87.5	100	62.5	87.5	100
13-HODE + 9-HODE	100	100	100	100	100	100	100	100	100	100	100	100	100	100	100	100	100	100	100	100	100	100	100	100	100	100	100	100	100	100	100	100	100	100	100	100	100	100	100	100	100	100	100	100	100	100	100	100	100	100
1-kestose	37.5	100	0	87.5	12.5	37.5	12.5	100	100	100	100	100	100	100	100	100	100	100	100	37.5	75	37.5	50	87.5	62.5	100	87.5	75	100	100	100	0	0	25	100	0	37.5	25	100	0	75	75	12.5	12.5	37.5	12.5	87.5	100	87.5	25
1-palmitoylglycerol (1-monopalmitin)	100	100	100	100	100	100	100	100	100	100	100	100	100	100	100	100	100	100	100	100	100	100	100	100	100	100	100	100	100	100	100	100	100	100	100	100	100	100	100	100	100	100	100	100	100	100	100	100	100	100
1-palmitoylglycerophosphoinositol*	100	100	100	100	100	100	100	100	100	100	100	100	100	100	100	100	100	100	100	100	100	100	100	100	100	100	100	100	100	100	100	100	100	100	100	100	100	100	100	100	100	100	100	100	100	100	100	100	100	100
1-stearoylglycerophosphoinositol	100	100	100	100	100	100	100	87.5	100	87.5	87.5	87.5	75	87.5	87.5	100	100	87.5	100	100	100	62.5	62.5	75	75	62.5	62.5	62.5	87.5	87.5	87.5	100	100	100	100	100	100	87.5	100	100	100	100	87.5	100	100	100	100	100	87.5	87.5
2-aminoadipate	100	100	100	87.5	87.5	100	100	100	100	100	100	100	87.5	100	100	87.5	100	87.5	100	100	100	100	100	100	100	100	100	100	100	100	100	100	100	100	100	100	100	100	100	75	100	100	100	100	100	100	100	87.5	100	100
2'-deoxyadenosine	100	100	100	100	100	100	100	100	100	100	100	100	100	100	100	100	100	100	100	100	100	100	100	100	100	100	100	100	100	100	100	100	100	100	100	100	100	100	100	100	100	100	100	100	100	100	100	100	100	100
2-hydroxyadipate	100	100	100	87.5	100	100	87.5	87.5	100	100	100	100	100	87.5	87.5	100	87.5	100	100	100	87.5	75	100	87.5	87.5	87.5	100	100	100	100	100	100	100	100	100	100	100	100	87.5	100	100	100	100	100	87.5	100	100	100	100	100
2-hydroxyglutarate	100	100	100	100	100	100	100	100	100	100	100	100	100	100	100	100	100	100	100	100	100	100	100	100	100	100	100	100	100	100	100	100	100	100	100	100	100	100	100	100	100	100	100	100	100	100	100	100	100	100
2-hydroxypalmitate	100	100	100	100	100	100	100	100	100	100	100	100	100	100	100	100	100	100	100	100	100	100	100	100	100	100	100	100	100	100	100	100	100	100	100	100	100	100	100	100	100	100	100	100	100	100	100	100	100	100
2-hydroxystearate	75	100	100	100	87.5	75	75	100	87.5	62.5	75	100	100	87.5	100	87.5	100	100	75	62.5	75	87.5	62.5	87.5	50	75	37.5	75	62.5	75	87.5	87.5	100	100	75	100	50	75	75	100	75	75	87.5	87.5	87.5	87.5	75	100	87.5	62.5
2-isopropylmalate	12.5	37.5	75	87.5	25	75	25	75	50	75	50	62.5	62.5	37.5	37.5	75	62.5	50	50	25	62.5	12.5	62.5	25	50	0	50	25	25	50	75	37.5	75	62.5	87.5	75	25	50	37.5	50	75	75	62.5	25	62.5	62.5	75	75	37.5	62.5
2-palmitoylglycerol (2-monopalmitin)	0	87.5	25	12.5	37.5	12.5	25	25	50	25	37.5	37.5	50	50	25	37.5	25	12.5	25	12.5	37.5	50	50	37.5	25	37.5	75	25	50	25	0	12.5	25	37.5	37.5	12.5	50	37.5	25	50	50	50	50	62.5	37.5	50	37.5	62.5	50	37.5
3-deoxyoctulosonate	75	100	75	62.5	87.5	87.5	62.5	87.5	87.5	100	75	87.5	75	75	75	100	62.5	50	62.5	62.5	100	100	62.5	50	75	75	100	100	75	75	87.5	62.5	62.5	75	87.5	50	62.5	87.5	75	87.5	75	87.5	100	87.5	100	100	100	75	50	87.5
3-methyl-2-oxovalerate	100	75	87.5	100	75	75	100	87.5	62.5	87.5	50	50	62.5	87.5	62.5	75	87.5	62.5	100	100	87.5	50	50	50	75	87.5	62.5	87.5	75	87.5	62.5	100	50	100	100	37.5	100	100	100	87.5	87.5	100	87.5	75	100	62.5	75	87.5	75	100
4-acetamidobutanoate	87.5	100	100	100	62.5	87.5	100	75	100	100	100	75	62.5	87.5	100	87.5	100	75	87.5	87.5	100	100	100	87.5	100	75	50	62.5	75	75	62.5	100	100	87.5	75	100	75	100	100	87.5	75	75	75	100	100	100	87.5	87.5	87.5	100
4-guanidinobutanoate	100	100	100	100	100	100	100	100	100	100	100	100	100	100	100	100	100	100	100	100	100	100	100	100	100	100	100	100	100	100	100	100	100	100	100	100	100	100	100	100	100	100	100	100	100	100	100	100	100	100
4-hydroxycinnamate	62.5	75	0	37.5	37.5	62.5	75	87.5	62.5	75	100	62.5	50	75	75	87.5	87.5	62.5	75	50	62.5	50	87.5	87.5	50	37.5	75	100	75	87.5	75	87.5	12.5	62.5	37.5	0	25	100	87.5	37.5	0	75	37.5	0	12.5	0	12.5	87.5	50	50
4-methyl-2-oxopentanoate	100	100	100	100	100	100	100	100	100	100	100	100	100	100	100	100	100	100	100	100	100	100	87.5	100	100	100	100	100	87.5	100	100	100	62.5	100	100	87.5	100	100	100	87.5	100	100	100	100	100	87.5	87.5	100	100	100
5-oxoproline	100	100	100	100	100	100	100	100	100	100	100	100	100	100	100	100	100	100	100	100	100	100	100	100	100	100	100	100	100	100	100	100	100	100	100	100	100	100	100	100	100	100	100	100	100	100	100	100	100	100
adenine	100	100	100	100	100	100	100	100	100	100	100	100	100	100	100	100	100	100	100	100	100	100	100	100	100	100	100	100	100	100	100	100	100	100	100	100	100	100	100	100	100	100	100	100	100	100	100	100	100	100
adenosine	100	100	100	100	100	100	100	100	100	100	100	100	100	100	100	100	100	100	100	100	100	100	100	100	100	100	100	100	100	100	100	100	100	100	100	100	100	100	100	100	100	100	100	100	100	100	100	100	100	100
adenosine 3'-monophosphate (3'-AMP)	87.5	100	100	100	100	100	100	100	100	100	100	100	100	100	100	100	100	100	100	100	87.5	100	100	100	100	100	100	100	87.5	100	100	100	100	100	100	100	100	87.5	100	100	100	100	100	100	100	100	100	100	100	100
adenosine 5'-monophosphate (AMP)	100	100	100	100	100	100	100	100	100	100	100	100	100	100	100	100	100	100	100	100	100	100	100	100	100	100	100	100	100	100	100	100	100	100	100	100	100	100	100	100	100	100	100	100	100	100	100	100	100	100
adenosine-2',3'-cyclic monophosphate	12.5	12.5	37.5	75	25	62.5	100	100	87.5	62.5	75	87.5	100	87.5	87.5	87.5	87.5	87.5	87.5	62.5	37.5	50	50	75	50	100	62.5	62.5	87.5	87.5	75	37.5	62.5	12.5	0	50	12.5	0	37.5	75	75	25	50	100	12.5	0	75	12.5	50	75
agmatine	0	12.5	62.5	62.5	25	37.5	75	100	75	37.5	50	62.5	100	75	75	87.5	87.5	100	25	87.5	50	87.5	50	75	50	75	50	50	62.5	75	62.5	75	0	25	25	62.5	37.5	37.5	50	50	37.5	25	50	100	12.5	25	75	0	37.5	12.5
alanine	100	100	100	100	100	100	100	100	100	100	100	100	100	100	100	100	100	100	100	100	100	100	100	100	100	100	100	100	100	100	100	100	100	100	100	100	100	100	100	100	100	100	100	100	100	100	100	100	100	100
alanylleucine	87.5	87.5	87.5	87.5	25	100	50	100	87.5	87.5	87.5	87.5	100	100	100	87.5	87.5	62.5	37.5	87.5	100	62.5	62.5	87.5	100	37.5	75	50	87.5	37.5	62.5	87.5	87.5	75	100	100	62.5	100	100	87.5	100	62.5	100	100	75	75	100	87.5	62.5	100
allantoic acid	25	12.5	100	12.5	50	87.5	25	50	0	12.5	25	12.5	87.5	50	25	75	25	50	0	0	37.5	12.5	37.5	12.5	37.5	25	25	25	12.5	25	50	50	75	25	75	75	12.5	12.5	0	50	87.5	12.5	62.5	100	37.5	100	25	0	25	50
allantoin	87.5	100	100	87.5	75	100	87.5	100	100	100	87.5	87.5	100	100	100	100	100	87.5	87.5	75	100	100	87.5	87.5	100	87.5	100	87.5	100	87.5	87.5	100	100	100	100	100	75	87.5	50	100	100	100	87.5	100	100	100	100	87.5	87.5	87.5
alpha-glutamylglutamate	100	100	100	100	87.5	100	100	100	100	100	100	100	100	100	100	100	100	100	100	100	87.5	100	100	100	100	87.5	100	100	100	100	100	100	100	100	100	100	100	100	100	100	100	100	100	100	100	100	100	100	87.5	100
alpha-ketoglutarate	87.5	87.5	75	100	87.5	100	100	100	100	100	87.5	100	100	100	87.5	87.5	100	100	100	100	100	100	87.5	100	100	87.5	75	100	100	100	100	87.5	100	87.5	75	100	100	100	87.5	87.5	100	75	100	100	100	75	100	100	100	87.5
arabitol	100	100	87.5	100	100	75	62.5	100	62.5	100	75	87.5	100	100	62.5	100	75	75	100	100	100	87.5	87.5	100	100	87.5	87.5	75	75	100	75	100	62.5	100	75	100	87.5	100	75	75	87.5	100	75	87.5	100	100	87.5	87.5	87.5	87.5
arabonate	100	100	100	100	100	100	100	100	100	100	100	100	100	100	100	100	100	100	100	100	100	100	100	100	87.5	100	100	100	100	100	100	100	100	100	100	100	100	100	100	100	100	100	100	100	100	100	100	100	100	100
arginine	100	100	100	100	100	100	100	100	100	100	100	100	100	100	100	100	100	100	100	100	100	100	100	100	100	100	100	100	100	100	100	100	100	100	100	100	100	100	100	100	100	100	100	100	100	100	100	100	100	100
asparagine	100	100	100	100	100	100	100	100	100	100	100	100	100	100	100	100	100	100	100	100	100	100	100	100	100	100	100	100	100	100	100	100	100	100	100	100	100	100	100	100	100	100	100	100	100	100	100	100	100	100
aspartate	100	100	100	100	100	100	100	100	100	100	100	100	100	100	100	100	100	100	100	100	100	100	100	100	100	100	100	100	100	100	100	100	100	100	100	100	100	100	100	100	100	100	100	100	100	100	100	100	100	100
azelate (nonanedioate)	25	50	62.5	12.5	37.5	37.5	25	37.5	12.5	37.5	25	37.5	62.5	12.5	37.5	87.5	37.5	50	25	12.5	75	37.5	37.5	0	0	12.5	0	25	37.5	37.5	12.5	62.5	75	75	50	62.5	50	25	75	62.5	50	50	37.5	50	50	75	87.5	25	50	50
beta-alanine	100	100	100	100	100	100	100	100	100	100	100	100	100	100	100	100	100	100	100	100	100	100	100	100	100	100	100	100	100	100	100	100	100	100	100	100	87.5	100	100	100	100	100	100	100	100	100	100	100	100	100
beta-sitosterol	100	100	100	100	100	100	100	100	100	100	100	100	100	100	100	100	100	100	100	100	100	100	100	75	100	100	87.5	100	87.5	100	100	100	100	100	100	100	100	100	100	100	100	100	87.5	100	100	100	100	100	100	100
campesterol	75	87.5	87.5	75	75	75	50	75	62.5	75	62.5	100	87.5	75	100	100	87.5	75	87.5	62.5	75	87.5	87.5	87.5	75	100	75	87.5	75	75	75	75	75	100	87.5	87.5	87.5	75	50	87.5	62.5	75	75	50	62.5	75	75	87.5	75	62.5
caproate (6:0)	100	100	100	100	100	100	100	100	100	100	100	100	100	100	100	100	100	100	100	100	100	100	100	100	100	100	100	100	100	100	100	100	100	100	100	100	100	100	100	100	100	100	100	100	100	100	100	100	100	100
chiro-inositol	100	100	100	100	100	100	100	100	100	100	100	100	100	100	100	100	100	100	100	100	100	100	100	100	100	100	100	100	100	100	100	100	100	100	100	100	100	100	100	100	100	100	100	100	100	100	100	100	100	100
choline phosphate	100	100	100	100	100	100	100	100	100	100	100	100	100	100	100	100	100	100	100	100	100	100	100	100	100	100	100	100	100	100	100	100	100	100	100	100	100	100	100	100	100	100	100	100	100	100	100	100	100	100
cis-aconitate	100	100	100	100	100	100	100	100	100	100	100	100	100	100	100	100	100	100	100	100	100	100	100	100	100	100	100	100	100	100	100	100	100	100	100	100	100	100	100	100	100	100	100	100	100	100	100	100	100	100
citramalate	100	100	87.5	100	87.5	100	87.5	100	100	100	100	100	100	100	100	100	100	87.5	87.5	100	100	87.5	100	100	100	100	100	100	100	100	100	100	100	100	100	100	100	87.5	100	100	100	100	100	100	87.5	100	100	87.5	100	100
citrate	100	100	100	100	100	100	100	100	100	100	100	100	100	100	100	100	100	100	100	100	100	100	100	100	100	100	100	100	100	100	100	100	100	100	100	100	100	100	100	100	100	100	100	100	100	100	100	100	100	100
citrulline	50	37.5	75	62.5	75	100	75	100	87.5	62.5	50	87.5	100	75	100	100	50	100	75	37.5	75	62.5	87.5	25	87.5	62.5	87.5	75	100	62.5	50	50	75	25	100	100	25	25	75	62.5	100	87.5	87.5	100	75	12.5	75	87.5	12.5	62.5
cysteine	100	100	100	87.5	100	62.5	100	100	75	87.5	75	100	100	100	87.5	87.5	87.5	100	100	87.5	100	100	100	100	100	100	100	100	100	100	100	100	100	100	75	100	100	87.5	87.5	100	100	100	62.5	100	75	62.5	87.5	87.5	87.5	87.5
cytidine	100	100	100	100	100	100	100	100	100	100	100	100	100	100	100	100	100	100	100	100	100	100	100	100	100	100	100	100	100	100	100	100	100	100	100	100	100	100	100	100	100	100	100	100	100	100	100	100	100	100
cytidine-3'-monophosphate (3'-CMP)	62.5	87.5	62.5	62.5	50	100	37.5	62.5	37.5	75	25	50	75	50	62.5	37.5	37.5	0	37.5	12.5	75	37.5	12.5	37.5	25	37.5	37.5	62.5	25	50	25	62.5	87.5	75	50	62.5	87.5	50	100	62.5	87.5	62.5	75	62.5	75	75	62.5	75	75	37.5
cytosine	100	100	100	100	100	100	62.5	100	100	62.5	100	100	100	100	100	100	75	100	100	100	100	87.5	100	87.5	75	75	87.5	87.5	87.5	87.5	87.5	87.5	100	100	100	100	62.5	100	100	100	100	100	100	100	87.5	100	100	100	100	87.5
daidzein	100	100	100	100	100	100	100	100	100	100	100	100	100	100	100	100	100	100	100	100	100	100	100	100	100	100	100	100	100	100	100	100	100	100	100	100	100	100	100	100	100	100	100	100	100	100	100	100	100	100
daidzin	100	100	100	100	100	100	100	100	100	100	100	100	100	100	100	100	100	100	100	100	100	100	100	100	100	100	100	100	100	100	100	100	87.5	100	100	100	100	100	100	100	75	100	100	87.5	100	100	100	100	100	100
delta-tocopherol	62.5	100	50	75	62.5	50	75	37.5	75	62.5	62.5	75	62.5	75	75	50	37.5	37.5	50	37.5	62.5	50	37.5	50	62.5	62.5	75	62.5	75	50	75	100	62.5	87.5	75	50	62.5	87.5	0	50	62.5	62.5	62.5	62.5	87.5	25	75	75	75	75
dihydrokaempferol	25	12.5	50	62.5	50	12.5	62.5	12.5	100	87.5	37.5	87.5	50	100	100	62.5	62.5	50	87.5	75	87.5	75	62.5	25	75	37.5	87.5	62.5	50	75	87.5	12.5	50	0	37.5	75	75	100	100	25	12.5	50	75	0	12.5	37.5	62.5	37.5	75	12.5
dimethylarginine (SDMA + ADMA)	87.5	100	100	87.5	100	100	100	100	62.5	100	87.5	62.5	100	75	100	100	100	87.5	75	75	100	87.5	75	75	100	75	75	87.5	87.5	100	87.5	100	100	87.5	100	62.5	75	100	75	100	100	100	100	87.5	87.5	87.5	75	87.5	75	50
ectoine	75	100	100	37.5	100	100	100	100	100	100	100	100	100	100	100	100	100	75	87.5	100	100	100	100	100	100	100	100	100	100	100	100	100	100	100	100	100	100	100	87.5	87.5	100	100	100	100	100	100	100	62.5	100	100
erythritol	100	100	100	100	75	87.5	75	100	100	100	100	100	100	100	100	100	87.5	100	100	87.5	100	100	100	87.5	100	100	100	100	100	100	100	100	100	100	87.5	87.5	62.5	100	100	100	100	100	75	100	87.5	100	87.5	100	100	87.5
erythronate*	62.5	100	62.5	62.5	75	87.5	75	75	75	62.5	25	100	100	62.5	100	75	87.5	37.5	50	37.5	87.5	87.5	62.5	50	50	62.5	62.5	75	50	62.5	50	100	62.5	100	75	87.5	37.5	87.5	75	50	100	62.5	75	62.5	62.5	87.5	100	37.5	100	87.5
ethanolamine	100	100	100	100	100	100	100	100	100	100	100	100	100	100	100	100	100	100	100	100	100	100	100	100	100	100	100	100	100	100	100	100	100	100	100	100	100	100	100	100	100	100	100	100	100	100	100	100	100	100
fructose	100	100	100	100	100	100	100	100	100	100	100	100	100	100	100	100	100	100	100	100	100	100	100	100	100	100	100	100	100	100	100	100	100	100	100	100	100	100	100	100	100	100	100	100	100	100	100	100	100	100
fumarate	100	100	100	100	100	100	100	100	100	100	100	100	100	100	100	100	100	100	100	100	100	100	100	100	100	100	100	100	100	100	100	100	100	100	100	100	100	100	100	100	100	100	100	100	100	100	100	100	100	100
galactarate (mucic acid)	100	100	100	100	100	100	100	100	100	100	100	100	100	100	100	100	100	100	100	100	100	100	100	100	100	100	100	100	100	100	100	100	100	100	100	100	100	100	100	100	100	100	100	100	100	100	100	100	100	100
galactinol	87.5	87.5	100	100	100	100	87.5	100	100	100	100	87.5	87.5	100	75	100	100	100	100	100	100	100	75	62.5	100	87.5	87.5	75	87.5	100	100	100	100	100	100	100	87.5	100	100	87.5	100	87.5	100	100	100	62.5	75	75	75	100
galactitol (dulcitol)	75	75	50	50	50	75	50	37.5	62.5	62.5	25	25	50	75	50	87.5	50	12.5	62.5	50	62.5	75	62.5	50	100	87.5	100	87.5	62.5	50	50	75	100	87.5	62.5	75	50	37.5	75	37.5	75	100	62.5	62.5	37.5	87.5	75	75	87.5	25
galactose	100	100	100	100	100	100	100	100	100	100	100	100	100	100	100	100	100	100	100	100	100	100	100	100	100	100	100	100	100	100	100	100	100	100	100	100	100	100	100	100	100	100	100	100	100	100	100	100	100	100
gamma-aminobutyrate (GABA)	100	100	100	100	100	100	100	100	100	100	100	100	100	100	100	100	100	100	100	100	100	100	100	100	100	100	100	100	100	100	100	100	100	100	100	100	100	100	100	100	100	100	100	100	100	100	100	100	100	100
gamma-glutamylglutamate	100	100	100	100	100	100	100	100	100	100	100	100	100	100	100	100	100	100	100	100	100	100	100	100	100	100	100	100	100	100	100	100	100	100	100	100	100	100	100	100	100	100	100	100	100	100	100	100	100	100
gamma-glutamylglutamine	100	100	100	100	100	100	100	100	100	100	100	100	100	100	100	100	100	100	100	100	100	100	100	100	100	100	100	100	100	100	100	100	100	100	100	100	100	100	100	100	100	100	100	100	100	100	100	100	100	100
gamma-glutamylhistidine	100	100	100	100	100	100	100	100	100	100	100	100	100	100	100	100	100	100	100	100	100	100	100	100	100	100	100	100	100	100	100	100	100	100	100	100	100	100	100	100	100	100	100	100	100	100	100	100	100	100
gamma-glutamylisoleucine*	100	100	100	100	100	100	100	100	100	100	100	100	100	100	100	100	100	100	100	100	100	100	100	100	100	100	100	100	100	100	100	100	100	100	100	100	100	100	100	100	100	100	100	100	100	100	100	100	100	100
gamma-glutamylmethionine	37.5	100	100	62.5	75	75	87.5	25	50	25	25	37.5	25	12.5	50	37.5	37.5	25	75	62.5	62.5	87.5	50	50	37.5	25	25	37.5	12.5	12.5	62.5	87.5	75	75	87.5	62.5	75	87.5	62.5	87.5	100	25	87.5	100	100	100	62.5	37.5	37.5	75
gamma-glutamylphenylalanine	100	100	100	100	100	100	100	100	100	100	100	100	100	100	100	100	100	100	100	100	100	100	100	100	100	100	100	100	100	100	100	100	100	100	100	100	100	100	100	100	100	100	100	100	100	100	100	100	100	100
gamma-glutamyltryptophan	100	100	100	100	100	100	100	100	100	100	100	100	100	100	100	100	100	100	100	100	100	100	100	100	100	100	100	100	100	100	100	100	100	100	100	100	100	100	100	100	100	100	100	100	100	100	100	100	100	100
gamma-glutamyltyrosine	100	100	100	100	100	100	100	100	100	100	100	100	100	100	100	100	100	100	100	100	100	100	100	100	100	100	100	100	100	100	100	100	100	100	100	100	100	100	100	100	100	100	100	100	100	100	100	100	100	100
gamma-glutamylvaline	100	100	100	100	100	100	100	100	87.5	100	100	87.5	100	100	87.5	100	100	100	100	100	100	100	100	100	100	100	100	100	100	87.5	100	100	100	100	100	100	100	100	100	100	100	100	100	100	100	100	100	100	100	100
gamma-tocopherol	62.5	87.5	0	0	12.5	50	12.5	0	12.5	25	25	12.5	12.5	0	25	0	12.5	0	0	37.5	12.5	37.5	0	0	12.5	12.5	0	0	12.5	0	12.5	75	50	62.5	37.5	25	37.5	37.5	62.5	25	12.5	25	25	50	50	50	37.5	25	25	50
genistein	100	100	100	100	100	100	100	100	100	100	100	100	100	100	100	100	100	100	100	100	100	100	100	100	100	100	100	100	100	100	100	100	100	100	100	100	100	100	100	100	100	100	100	100	100	100	100	100	100	100
genistin	100	100	100	100	100	100	100	100	100	100	100	100	100	100	100	100	100	100	100	100	100	100	100	100	100	100	100	100	100	100	100	100	100	100	100	100	100	100	100	100	75	100	100	87.5	100	100	100	100	100	100
glucarate (saccharate)	100	100	100	100	100	100	100	100	100	100	100	100	100	100	100	100	100	100	100	100	100	100	100	100	100	100	100	100	100	100	100	100	100	100	100	100	100	100	100	100	100	100	100	100	100	100	100	100	100	100
glucarate 1,4-lactone	100	100	100	100	100	100	100	100	100	87.5	100	100	87.5	100	100	100	100	100	87.5	100	100	100	100	100	100	100	100	100	100	100	100	100	100	100	100	100	100	100	100	100	100	100	100	100	100	100	100	100	100	100
gluconate	87.5	100	100	100	100	87.5	100	100	100	100	100	100	100	100	100	100	100	100	100	100	100	100	100	100	100	100	100	100	100	100	100	100	100	100	100	100	100	100	100	100	100	100	87.5	100	100	100	100	100	87.5	100
glucose	100	100	100	100	100	100	100	100	100	100	100	100	100	100	100	100	100	100	100	100	100	100	100	100	100	100	100	100	100	100	100	100	100	100	100	100	100	100	100	100	100	100	100	100	100	100	100	100	100	100

BIOCHEMICAL	JACK	GM	L1	L2	L3	L4	L5	L6	L7	L8	L9	L10	L11	L12	L13	L14	L15	L16	L17	L18	L19	L20	L21	L22	L23	L24	L25	L26	L27	L28	L29	L30	L31	L32	L33	L34	L35	L36	L37	L38	L39	L40	L41	L42	L43	L44	L45	L46	L47	L48
glutamate	100	100	100	100	100	100	100	100	100	100	100	100	100	100	100	100	100	100	100	100	100	100	100	100	100	100	100	100	100	100	100	100	100	100	100	100	100	100	100	100	100	100	100	100	100	100	100	100	100	100
glutamine	100	100	100	100	100	100	100	100	100	100	100	100	100	100	100	100	100	100	100	87.5	100	100	100	100	100	100	100	100	100	100	100	100	100	100	100	100	100	87.5	100	100	100	100	100	100	100	100	87.5	100	100	87.5
glycerate	100	100	100	100	100	100	100	100	100	100	100	100	100	100	100	100	100	100	100	100	100	100	100	100	100	100	87.5	100	100	100	100	100	100	100	100	100	100	100	100	100	100	100	100	100	100	100	100	100	100	100
glycerol	100	100	100	100	100	100	100	100	100	100	100	100	100	100	100	100	100	100	100	100	100	100	100	100	100	100	100	100	100	100	100	100	100	100	100	100	100	100	100	100	100	100	100	100	100	100	100	100	100	100
glycerol 2-phosphate	87.5	100	75	87.5	75	87.5	100	100	100	100	100	100	100	100	100	100	100	100	100	87.5	100	100	100	100	87.5	87.5	87.5	100	87.5	100	100	100	100	75	75	100	87.5	100	75	100	87.5	87.5	75	100	87.5	100	100	100	100	87.5
glycerol 3-phosphate (G3P)	100	100	100	100	100	100	100	100	100	100	100	100	100	100	100	100	100	100	100	100	100	100	100	100	100	100	100	100	100	100	100	100	100	100	100	100	100	100	100	100	100	100	100	100	100	100	100	100	100	100
glycerophosphorylcholine (GPC)	100	100	100	100	100	100	100	100	100	100	100	100	100	100	100	100	100	100	100	100	100	100	100	100	100	100	100	100	100	100	100	100	100	100	100	100	100	100	100	100	100	100	100	100	100	100	100	100	100	100
glycine	100	100	100	100	100	100	100	100	100	100	100	100	100	100	100	100	100	100	100	100	100	100	100	100	100	100	100	100	100	100	100	100	100	100	100	100	100	100	100	100	100	100	100	100	100	100	100	100	100	100
glycitein	100	100	100	100	100	100	100	100	100	100	100	100	100	100	100	100	100	100	100	100	100	100	100	100	100	100	87.5	100	100	100	100	100	100	100	100	100	100	100	100	100	75	100	100	100	100	100	100	100	100	100
glycitin	100	100	100	100	100	100	100	100	100	100	100	100	100	100	100	100	100	100	100	100	100	100	100	100	100	100	100	100	100	100	100	100	100	100	100	100	100	100	100	100	87.5	100	100	100	100	100	100	100	100	100
glycyltyrosine	100	100	100	75	75	100	62.5	100	100	75	100	87.5	87.5	87.5	87.5	100	87.5	62.5	62.5	62.5	100	87.5	75	75	62.5	50	75	87.5	87.5	75	75	100	87.5	100	100	75	75	100	100	100	100	87.5	87.5	100	87.5	75	87.5	100	100	87.5
guanine	75	87.5	100	100	87.5	75	100	100	100	100	100	100	87.5	100	100	100	100	87.5	87.5	75	75	100	100	87.5	75	87.5	100	75	87.5	100	87.5	87.5	87.5	100	87.5	75	87.5	87.5	100	100	100	100	100	100	100	100	100	100	87.5	87.5
guanosine	100	100	100	100	100	100	100	100	100	100	100	100	100	100	100	100	100	100	100	100	100	100	100	100	100	100	100	100	100	100	100	100	100	100	100	100	100	100	100	100	100	100	100	100	100	100	100	100	100	100
guanosine-2',3'-cyclic monophosphate	12.5	0	12.5	75	25	50	87.5	100	75	75	75	87.5	87.5	75	100	87.5	87.5	100	87.5	75	37.5	62.5	50	75	50	100	62.5	62.5	87.5	87.5	87.5	37.5	50	12.5	12.5	50	12.5	0	37.5	62.5	62.5	25	50	100	12.5	0	62.5	12.5	37.5	75
gulono-1,4-lactone	100	100	100	100	100	100	100	100	100	100	100	100	100	100	100	100	100	100	100	100	100	100	100	100	87.5	100	100	100	100	100	100	100	100	100	100	100	100	100	100	100	100	100	100	100	100	100	100	100	100	100
histidine	100	100	100	100	100	100	100	100	100	100	100	100	100	100	100	100	100	100	100	100	100	100	100	100	100	100	100	100	100	100	100	100	100	100	100	100	100	100	100	100	100	100	100	100	100	100	100	100	100	100
homoserine	75	62.5	100	62.5	87.5	75	100	100	100	100	87.5	87.5	100	100	87.5	100	87.5	87.5	100	75	100	87.5	100	75	100	87.5	75	87.5	62.5	87.5	75	100	100	75	100	100	37.5	100	100	87.5	87.5	75	87.5	75	75	87.5	87.5	75	62.5	62.5
inositol 1-phosphate (I1P)	100	100	100	100	100	100	100	100	100	100	100	100	100	100	100	100	100	100	100	100	100	100	100	100	100	100	100	100	100	100	100	100	100	100	100	100	100	100	100	100	100	100	100	100	100	100	100	100	100	100
inositol 2-phosphate (I2P)	87.5	87.5	37.5	75	62.5	75	100	87.5	87.5	75	62.5	50	87.5	75	87.5	75	62.5	100	87.5	87.5	100	100	87.5	25	62.5	62.5	50	37.5	37.5	62.5	62.5	75	75	62.5	100	75	62.5	75	100	100	75	25	75	75	62.5	50	87.5	62.5	75	87.5
isocitrate	100	100	100	100	100	100	100	100	100	100	100	100	100	100	100	100	100	100	100	100	100	100	100	100	100	100	100	100	100	100	100	100	100	100	100	100	100	100	100	100	100	100	100	100	100	100	100	100	100	100
isoleucine	100	100	100	100	100	100	100	100	100	100	100	100	100	100	100	100	100	100	100	100	100	100	100	100	100	100	100	100	100	100	100	100	100	100	100	100	100	100	100	100	100	100	100	100	100	100	100	100	100	100
leucine	100	100	100	100	100	100	100	100	100	100	100	100	100	100	100	100	100	100	100	100	100	100	100	100	100	100	100	100	100	100	100	100	100	100	100	100	100	100	100	100	100	100	100	100	100	100	100	100	100	100
linoleate (18:2n6)	100	100	100	100	100	100	100	100	100	100	100	100	100	100	100	100	100	100	100	100	100	100	100	100	100	100	100	100	100	100	100	100	100	100	100	100	100	100	100	100	100	100	100	100	100	100	100	100	100	100
linolenate [alpha or gamma; (18:3n3 or 6)]	100	100	62.5	75	100	87.5	37.5	100	75	62.5	75	25	75	62.5	50	75	37.5	37.5	62.5	87.5	75	75	50	25	37.5	75	25	75	75	87.5	37.5	87.5	37.5	87.5	62.5	75	87.5	100	100	62.5	75	75	75	75	100	100	75	100	100	75
lysine	100	100	100	100	100	100	100	100	100	100	100	100	100	100	100	100	100	100	100	100	100	100	100	100	100	100	100	100	100	100	100	100	100	100	100	100	100	100	100	100	100	100	100	100	100	100	100	100	100	100
malate	100	100	100	100	100	100	100	100	100	100	100	100	100	100	100	100	100	100	100	100	100	100	100	100	100	100	100	100	100	100	100	100	100	100	100	100	100	100	100	100	100	100	100	100	100	100	100	100	100	100
malonate (propanedioate)	100	100	100	100	100	100	100	100	100	100	100	100	100	100	100	100	100	100	100	100	100	100	100	100	100	100	100	100	100	100	100	100	100	100	100	100	100	100	100	100	100	100	100	100	100	100	100	100	100	100
mannitol	100	100	100	100	100	100	100	100	100	100	100	100	100	100	100	100	100	100	100	100	100	100	100	100	100	100	100	100	100	100	100	100	100	100	100	100	100	100	100	100	100	100	100	100	100	100	100	100	100	100
methionine	100	100	100	100	100	100	100	100	100	100	100	100	100	100	100	100	100	100	100	100	100	100	100	100	100	100	100	100	100	100	100	100	100	100	100	100	100	100	100	100	100	100	100	100	100	100	100	100	100	100
methionine sulfoxide	100	100	100	100	100	100	100	100	100	100	100	100	100	100	100	100	100	100	100	100	100	100	100	100	100	100	100	100	100	100	100	100	100	100	100	100	100	100	100	100	100	100	100	100	100	100	100	100	100	100
methylphosphate	87.5	100	87.5	100	87.5	100	100	100	100	100	100	100	100	100	100	100	100	100	87.5	100	75	100	100	100	87.5	100	87.5	100	100	100	100	100	87.5	100	87.5	100	87.5	100	100	100	100	100	87.5	100	100	87.5	100	100	100	100
methylsuccinate	25	62.5	25	50	12.5	62.5	25	37.5	25	0	37.5	62.5	12.5	25	37.5	62.5	12.5	12.5	50	25	25	62.5	0	37.5	25	62.5	37.5	25	50	37.5	37.5	37.5	25	50	62.5	50	0	37.5	0	12.5	75	37.5	62.5	75	37.5	0	50	25	0	25
myo-inositol	100	100	100	100	100	100	100	100	100	100	100	100	100	100	100	100	100	100	100	100	100	100	100	100	100	100	100	100	100	100	100	100	100	100	100	100	100	100	100	100	100	100	100	100	100	100	100	100	100	100
myo-inositol hexakisphosphate	100	100	100	100	100	100	100	100	100	100	100	100	100	100	100	100	100	100	100	100	100	100	100	100	100	100	100	100	100	100	100	100	100	100	100	100	100	100	100	100	100	100	100	100	100	100	100	100	87.5	100
N6-acetyllysine	25	37.5	25	75	75	50	50	12.5	62.5	62.5	37.5	37.5	37.5	25	50	62.5	25	50	62.5	62.5	87.5	87.5	87.5	87.5	75	50	75	75	62.5	87.5	62.5	75	37.5	62.5	50	37.5	25	87.5	87.5	25	25	100	62.5	25	50	100	37.5	87.5	37.5	50
N-acetylornithine	100	100	100	100	100	100	100	100	100	100	100	100	100	100	100	100	100	100	100	100	100	100	100	100	100	100	100	100	100	100	100	100	100	100	100	100	100	100	100	100	100	100	100	100	100	100	100	100	100	100
naringenin	100	100	100	100	100	100	100	100	100	100	100	100	100	100	100	100	100	100	100	100	100	100	100	100	100	100	100	100	100	100	100	100	100	100	100	100	100	100	100	100	87.5	100	100	87.5	100	100	100	100	100	100
naringenin-7-O-glucoside	12.5	50	0	62.5	25	12.5	87.5	62.5	62.5	37.5	87.5	50	37.5	75	50	12.5	50	12.5	37.5	12.5	50	50	0	37.5	37.5	37.5	50	87.5	75	25	62.5	25	0	12.5	25	0	37.5	87.5	87.5	0	0	12.5	0	0	12.5	25	0	25	100	12.5
N-carbamoylaspartate	25	0	0	0	0	12.5	0	50	75	87.5	75	87.5	87.5	100	100	75	62.5	62.5	100	12.5	37.5	25	12.5	25	37.5	0	0	12.5	62.5	0	12.5	37.5	12.5	12.5	25	25	0	0	0	0	12.5	12.5	12.5	50	25	12.5	0	0	0	37.5
nicotinamide ribonucleotide (NMN)	100	100	100	100	100	100	100	100	100	100	100	100	100	100	100	100	100	100	100	100	100	100	100	100	100	100	100	100	100	100	100	100	100	100	100	100	100	100	100	100	100	100	100	100	100	100	100	100	100	100
nicotinamide riboside*	100	100	100	100	100	100	100	100	100	100	100	100	100	100	100	100	100	100	100	100	100	100	100	100	100	100	100	100	100	100	100	100	100	100	100	100	100	100	100	100	100	100	100	100	100	100	100	100	100	100
nicotinate ribonucleoside*	100	100	100	100	100	100	100	100	100	100	100	100	100	100	100	100	100	100	100	100	100	100	100	100	100	100	100	100	100	100	100	100	100	100	100	100	100	100	100	100	100	100	100	100	100	100	100	100	100	100
oleate (18:1n9)	100	100	100	100	100	100	100	100	100	100	100	100	100	100	100	100	100	100	100	100	100	100	100	100	100	100	100	100	100	100	100	100	100	100	100	100	100	100	100	100	100	100	100	100	100	100	100	100	100	100
pantothenate	100	100	100	100	100	100	100	100	100	100	100	100	100	100	100	100	100	100	100	100	100	100	100	100	100	100	100	100	100	100	100	100	100	100	100	100	100	100	100	100	100	100	100	100	100	100	100	100	100	100
phenylalanine	100	100	100	100	100	100	100	100	100	100	100	100	100	100	100	100	100	100	100	100	100	100	100	100	100	100	100	100	100	100	100	100	100	100	100	100	100	100	100	100	100	100	100	100	100	100	100	100	100	100
phosphate	100	100	100	100	100	100	100	100	100	100	100	100	100	100	100	100	100	100	100	100	100	100	100	100	100	100	100	100	100	100	100	100	100	100	100	100	100	100	100	100	100	100	100	100	100	100	100	100	100	100
p-hydroxybenzaldehyde	12.5	12.5	50	62.5	37.5	12.5	50	37.5	50	25	87.5	75	25	62.5	50	87.5	37.5	62.5	62.5	62.5	37.5	12.5	62.5	62.5	50	12.5	50	62.5	0	12.5	12.5	12.5	37.5	0	12.5	25	62.5	0	25	25	37.5	62.5	87.5	62.5	12.5	12.5	12.5	12.5	37.5	12.5
pinitol	100	100	100	100	100	100	100	100	100	100	100	100	100	100	100	100	100	100	100	100	100	100	100	100	100	100	100	100	100	100	100	100	100	100	100	100	100	100	100	100	100	100	100	100	100	100	100	100	100	100
pipecolate	100	100	100	100	100	100	100	100	100	100	100	100	100	100	100	100	100	100	100	100	100	100	100	100	100	100	100	100	100	100	100	100	100	100	100	100	100	100	100	100	100	100	100	100	100	100	100	100	100	100
proline	100	100	100	100	100	100	100	100	100	100	100	100	100	100	100	100	100	100	100	100	100	100	100	100	100	100	100	100	100	100	100	100	100	100	100	100	100	100	100	100	100	100	100	100	100	100	100	100	100	100
putrescine	75	50	75	100	87.5	75	100	100	100	100	87.5	100	100	100	100	100	87.5	100	100	87.5	100	100	87.5	100	100	100	100	87.5	100	100	100	62.5	50	62.5	75	87.5	62.5	100	62.5	100	62.5	100	62.5	100	75	50	100	37.5	87.5	87.5
pyridoxate	100	100	100	62.5	100	100	100	62.5	100	100	87.5	50	50	75	87.5	50	75	50	100	87.5	100	100	100	100	100	75	75	100	87.5	75	50	100	100	100	100	100	87.5	100	87.5	100	100	100	100	100	100	100	87.5	87.5	87.5	87.5
pyroglutamine*	100	100	100	100	100	100	100	100	100	100	100	100	100	100	100	100	100	100	100	100	100	100	100	100	100	100	100	100	100	100	100	100	100	100	100	100	100	100	100	100	100	100	100	100	100	100	100	100	100	100
pyroglutamylglutamine	62.5	100	100	62.5	87.5	100	62.5	75	100	62.5	75	50	37.5	37.5	75	50	37.5	25	37.5	75	100	75	50	75	37.5	37.5	50	50	62.5	62.5	25	87.5	75	100	75	62.5	37.5	87.5	100	62.5	87.5	87.5	87.5	50	100	62.5	50	62.5	50	37.5
pyruvate	100	100	100	100	100	100	100	100	100	100	100	100	100	100	100	100	100	100	100	100	100	100	100	100	100	100	100	100	100	100	100	100	100	100	100	100	100	100	100	100	100	100	100	100	100	100	100	100	100	100
raffinose	100	100	100	100	100	100	100	100	100	100	100	100	100	100	100	100	100	100	100	100	100	100	100	100	100	100	100	100	100	100	100	100	100	100	100	100	100	100	100	100	100	100	100	100	100	100	100	100	100	100
ribitol	37.5	87.5	25	100	37.5	25	62.5	87.5	100	100	100	100	100	100	100	87.5	100	75	100	50	87.5	100	100	100	100	87.5	87.5	100	100	100	50	75	37.5	62.5	12.5	87.5	25	62.5	50	62.5	50	87.5	25	50	37.5	100	75	87.5	87.5	50
ribose	100	100	100	100	100	100	100	100	100	100	100	100	100	100	100	100	100	100	100	100	100	100	100	100	100	100	100	100	100	100	100	100	100	100	100	100	100	100	100	100	100	100	100	100	100	100	100	100	100	100
scyllo-inositol	0	12.5	50	12.5	62.5	50	50	25	62.5	37.5	25	25	37.5	37.5	25	87.5	0	25	12.5	62.5	75	50	37.5	12.5	62.5	37.5	37.5	37.5	25	0	0	37.5	25	75	62.5	62.5	0	50	75	75	87.5	50	50	62.5	87.5	62.5	25	12.5	0	50
serine	100	100	100	100	100	100	100	100	100	100	100	100	100	100	100	100	100	100	100	100	100	100	100	100	100	100	100	100	100	100	100	100	100	100	100	100	100	100	100	100	100	100	100	100	100	100	100	100	100	100
sorbitol	100	100	100	100	87.5	100	100	100	100	100	100	100	100	100	100	100	100	100	100	100	100	100	100	100	100	100	100	100	100	100	100	100	100	100	100	100	100	100	100	100	100	100	100	100	100	100	100	100	100	100
spermidine	100	87.5	87.5	100	87.5	100	100	100	87.5	100	100	100	100	100	100	100	100	100	100	100	75	100	100	100	100	100	100	100	100	100	100	100	87.5	87.5	100	75	100	62.5	87.5	100	87.5	87.5	75	100	100	100	100	75	100	100
stachyose	100	100	100	100	100	100	100	100	100	100	100	100	100	100	100	100	100	100	100	100	100	100	100	100	100	100	100	100	100	100	100	100	100	100	100	100	100	100	100	100	100	100	100	100	100	100	100	100	100	100
stearamide	50	62.5	87.5	87.5	75	87.5	87.5	100	75	75	87.5	87.5	100	87.5	75	75	87.5	100	100	75	75	62.5	75	87.5	75	100	62.5	75	100	100	87.5	50	87.5	75	62.5	75	50	62.5	37.5	100	87.5	87.5	87.5	87.5	62.5	87.5	100	87.5	62.5	62.5
stigmasterol	100	100	100	100	87.5	100	87.5	100	87.5	100	87.5	100	87.5	100	100	100	100	87.5	100	100	100	100	100	87.5	87.5	87.5	75	100	87.5	75	87.5	100	100	100	87.5	87.5	100	87.5	87.5	100	100	87.5	100	100	100	100	100	100	87.5	100
succinate	100	100	100	100	100	100	100	100	100	100	100	100	100	100	100	100	100	87.5	100	100	100	100	100	100	100	100	100	100	100	100	100	100	100	100	100	100	100	100	100	100	100	100	100	100	100	100	100	100	100	100
sucrose	100	100	100	100	100	100	100	100	100	100	100	100	100	100	100	100	100	100	100	100	100	100	100	100	100	100	100	100	100	100	100	100	100	100	100	100	100	100	100	100	100	100	100	100	100	100	100	100	100	100
syringic acid	100	100	100	100	100	100	100	100	100	100	87.5	100	100	100	100	87.5	100	100	87.5	100	100	87.5	100	100	100	100	100	100	100	100	100	100	100	100	100	100	100	100	100	100	100	100	100	100	100	100	100	100	100	100
threitol	100	100	100	100	62.5	100	75	100	100	100	100	100	100	100	100	100	87.5	100	100	87.5	100	100	100	87.5	100	100	100	100	100	100	100	100	100	100	100	100	87.5	100	100	100	100	100	87.5	100	100	100	87.5	100	87.5	100
threonate	100	100	100	100	100	100	100	100	100	100	100	100	100	100	100	100	100	100	100	100	100	100	100	100	100	100	100	100	100	100	100	100	100	100	100	100	100	100	100	100	100	100	100	100	100	100	100	100	100	100
threonine	100	100	100	100	100	100	100	100	100	100	100	100	100	100	100	100	100	100	100	100	100	100	100	100	100	100	100	100	100	100	100	100	100	100	100	100	100	100	100	100	100	100	100	100	100	100	100	100	100	100
trans-4-hydroxyproline	25	62.5	25	25	37.5	25	12.5	50	25	50	25	37.5	37.5	62.5	37.5	50	37.5	62.5	37.5	37.5	75	87.5	37.5	50	50	12.5	25	75	75	12.5	87.5	37.5	62.5	37.5	25	50	12.5	50	37.5	37.5	62.5	25	50	37.5	37.5	75	25	62.5	12.5	25
trigonelline (N'-methylnicotinate)	100	100	100	100	100	100	100	100	100	100	100	100	100	100	100	100	100	100	100	100	100	100	100	100	100	100	100	100	100	100	100	100	100	100	100	100	100	100	100	100	100	100	100	100	100	100	100	100	100	100
tryptophan	100	100	100	100	100	100	100	100	100	100	100	100	100	100	100	100	100	100	100	100	100	100	100	100	100	100	100	100	100	100	100	100	100	100	100	100	100	100	100	100	100	100	100	100	100	100	100	100	100	100
tyrosine	100	100	100	100	100	100	100	100	100	100	100	100	100	100	100	100	100	100	100	100	100	100	100	100	100	100	100	100	100	100	100	100	100	100	100	100	100	100	100	100	100	100	100	100	100	100	100	100	100	100
uridine	100	100	100	100	100	100	100	100	100	100	100	100	100	100	100	100	100	100	100	100	100	100	100	100	100	100	100	100	100	100	100	100	100	100	100	100	100	100	100	100	100	100	100	100	100	100	100	100	100	100
uridine-2',3'-cyclic monophosphate	0	0	0	62.5	0	12.5	37.5	100	12.5	37.5	12.5	25	62.5	25	25	12.5	37.5	37.5	25	25	12.5	12.5	25	25	12.5	62.5	25	0	12.5	12.5	0	12.5	0	0	0	12.5	0	0	0	12.5	0	0	12.5	50	0	0	0	0	0	25
valine	100	100	100	100	100	100	100	100	100	100	100	100	100	100	100	100	100	100	100	100	100	100	100	100	100	100	100	100	100	100	100	100	100	100	100	100	100	100	100	100	100	100	100	100	100	100	100	100	100	100
valylglutamate	100	100	100	100	100	100	100	100	100	100	100	100	100	100	100	100	100	100	100	100	100	100	100	100	100	100	100	100	100	100	100	100	100	100	100	100	100	100	100	100	100	100	100	100	100	100	100	100	100	100
vanillate	100	100	100	100	100	100	100	100	100	100	100	100	100	100	100	100	100	100	100	100	100	100	100	100	100	100	100	100	100	100	100	100	100	100	100	100	100	100	100	100	100	100	100	100	100	100	100	100	100	100
verbascose	100	100	100	75	87.5	100	100	100	100	100	100	100	100	87.5	100	100	100	100	87.5	100	100	100	100	100	100	87.5	100	100	100	100	100	100	100	100	100	100	87.5	100	100	87.5	100	100	100	100	100	100	100	100	87.5	87.5
xanthosine	100	100	100	100	100	100	100	100	100	100	100	100	100	100	100	100	100	100	100	100	100	100	100	100	100	100	100	100	100	100	100	100	100	100	100	100	100	100	100	100	100	100	100	100	100	100	100	100	100	100
xylitol	87.5	100	100	100	87.5	87.5	87.5	100	100	100	100	100	100	100	100	100	100	87.5	100	100	100	100	100	100	100	100	100	87.5	75	100	100	100	100	100	87.5	100	87.5	100	100	100	100	100	100	100	100	100	87.5	100	100	100
xylonate	100	100	100	100	100	100	100	100	100	100	100	100	100	100	100	100	100	100	100	100	100	100	100	100	100	100	100	100	100	100	100	100	100	100	100	100	100	100	100	100	100	100	100	100	100	100	100	100	100	100

Supplemental Figure 1. Scatter/box-whisker plot comparisons for all compounds, arranged by biochemical pathway. Following the alphabetical reference list below, box-whisker plots showing relative values for all data (n=8) for the GM line (HPPD JACK), JACK, and the combined Conventional lines (WT) are displayed, grouped by biochemical pathway. The Y-axis represents log2 data scaled to the median detected value (median = 0). The box-whisker plot format shows the mean (+), median (horizontal line), and the quartile distribution of data points, such that the box represents the two central quartiles and the upper and lower whiskers represent the upper and lower quartiles, respectively. The MAX and MIN values define the top and bottom of the upper and lower quartiles. Imputed data points (if any) are included, and occur at the MIN value (lower whisker limit) for one or more of the plots.

BIOCHEMICAL	Super Pathway	Sub Pathway
**1,3-dihydroxyacetone**	Carbohydrate	Glycolysis
**13-HODE + 9-HODE**	Lipids	Oxylipins
**1-kestose**	Carbohydrate	Sucrose, glucose, fructose metabolism
**1-palmitoylglycerol (1-monopalmitin)**	Lipids	Glycerolipids
**1-palmitoylglycerophosphoinositol***	Lipids	Phospholipids
**1-stearoylglycerophosphoinositol**	Lipids	Lysolipids
**2-aminoadipate**	Amino acid	Aspartate family (OAA derived)
**2'-deoxyadenosine**	Nucleotide	Purine metabolism
**2-hydroxyadipate**	Lipids	Free fatty acid
**2-hydroxyglutarate**	Lipids	Free fatty acid
**2-hydroxypalmitate**	Lipids	Free fatty acid
**2-hydroxystearate**	Lipids	Free fatty acid
**2-isopropylmalate**	Amino acid	Branched Chain Amino Acids (pyruvate derived)
**2-palmitoylglycerol (2-monopalmitin)**	Lipids	Glycerolipids
**3-deoxyoctulosonate**	Carbohydrate	Sucrose, glucose, fructose metabolism
**3-methyl-2-oxovalerate**	Amino acid	Branched Chain Amino Acids (pyruvate derived)
**4-acetamidobutanoate**	Amino acid	Glutamate family (alpha-ketoglutarate derived)
**4-guanidinobutanoate**	Amino acid	Serine family (phosphoglycerate derived)
**4-hydroxycinnamate**	Amino acid	Aromatic amino acid metabolism (PEP derived)
**4-methyl-2-oxopentanoate**	Amino acid	Branched Chain Amino Acids (pyruvate derived)
**5-oxoproline**	Amino acid	Glutathione metabolism
**adenine**	Nucleotide	Purine metabolism
**adenosine**	Nucleotide	Purine metabolism
**adenosine 3'-monophosphate (3'-AMP)**	Nucleotide	Purine metabolism
**adenosine 5'-monophosphate (AMP)**	Nucleotide	Purine metabolism
**adenosine-2',3'-cyclic monophosphate**	Nucleotide	Purine metabolism
**agmatine**	Amino acid	Amines and polyamines
**alanine**	Amino acid	Aspartate family (OAA derived)
**alanylleucine**	Peptide	Dipeptide
**allantoic acid**	Nucleotide	Purine metabolism
**allantoin**	Nucleotide	Purine metabolism
**alpha-glutamylglutamate**	Peptide	Dipeptide
**alpha-ketoglutarate**	Carbohydrate	TCA cycle
**arabitol**	Carbohydrate	Amino sugar and nucleotide sugar
**arabonate**	Carbohydrate	Amino sugar and nucleotide sugar
**arginine**	Amino acid	Glutamate family (alpha-ketoglutarate derived)
**asparagine**	Amino acid	Aspartate family (OAA derived)
**aspartate**	Amino acid	Aspartate family (OAA derived)
**azelate (nonanedioate)**	Lipids	Free fatty acid
**beta-alanine**	Amino acid	Aspartate family (OAA derived)
**beta-sitosterol**	Lipids	Sterols
**campesterol**	Lipids	Sterols
**caproate (6:0)**	Lipids	Free fatty acid
**chiro-inositol**	Carbohydrate	Inositol metabolism
**choline phosphate**	Lipids	Choline metabolism
**cis-aconitate**	Carbohydrate	TCA cycle
**citramalate**	Carbohydrate	C5 branched dibasic acid metabolism
**citrate**	Carbohydrate	TCA cycle
**citrulline**	Amino acid	Glutamate family (alpha-ketoglutarate derived)
**cysteine**	Amino acid	Serine family (phosphoglycerate derived)
**cytidine**	Nucleotide	Pyrimidine metabolism
**cytidine-3'-monophosphate (3'-CMP)**	Nucleotide	Pyrimidine metabolism
**cytosine**	Nucleotide	Pyrimidine metabolism
**daidzein**	Secondary metabolism	Flavonoids
**daidzin**	Secondary metabolism	Flavonoids
**delta-tocopherol**	Cofactors, Prosthetic Groups, Electron Carriers	Tocopherol metabolism
**dihydrokaempferol**	Secondary metabolism	Flavonoids
**dimethylarginine (SDMA + ADMA)**	Amino acid	Glutamate family (alpha-ketoglutarate derived)
**ectoine**	Nucleotide	Pyrimidine metabolism
**erythritol**	Carbohydrate	Amino sugar and nucleotide sugar
**erythronate***	Carbohydrate	Amino sugar and nucleotide sugar
**ethanolamine**	Lipids	Choline metabolism
**fructose**	Carbohydrate	Sucrose, glucose, fructose metabolism
**fumarate**	Carbohydrate	TCA cycle
**galactarate (mucic acid)**	Secondary metabolism	Fatty acid and sugar derivatives
**galactinol**	Carbohydrate	Sucrose, glucose, fructose metabolism
**galactitol (dulcitol)**	Carbohydrate	Sucrose, glucose, fructose metabolism
**galactose**	Carbohydrate	Sucrose, glucose, fructose metabolism
**gamma-aminobutyrate (GABA)**	Amino acid	Glutamate family (alpha-ketoglutarate derived)
**gamma-glutamylglutamate**	Peptide	gamma-glutamyl
**gamma-glutamylglutamine**	Peptide	gamma-glutamyl
**gamma-glutamylhistidine**	Peptide	gamma-glutamyl
**gamma-glutamylisoleucine***	Peptide	gamma-glutamyl
**gamma-glutamylmethionine**	Peptide	gamma-glutamyl
**gamma-glutamylphenylalanine**	Peptide	gamma-glutamyl
**gamma-glutamyltryptophan**	Peptide	gamma-glutamyl
**gamma-glutamyltyrosine**	Peptide	gamma-glutamyl
**gamma-glutamylvaline**	Peptide	gamma-glutamyl
**gamma-tocopherol**	Cofactors, Prosthetic Groups, Electron Carriers	Tocopherol metabolism
**genistein**	Secondary metabolism	Flavonoids
**genistin**	Secondary metabolism	Flavonoids
**glucarate (saccharate)**	Cofactors, Prosthetic Groups, Electron Carriers	Ascorbate metabolism
**glucarate 1,4-lactone**	Carbohydrate	Sucrose, glucose, fructose metabolism
**gluconate**	Carbohydrate	Amino sugar and nucleotide sugar
**glucose**	Carbohydrate	Glycolysis

BIOCHEMICAL	Super Pathway	Sub Pathway
**glutamate**	Amino acid	Glutamate family (alpha-ketoglutarate derived)
**glutamine**	Amino acid	Glutamate family (alpha-ketoglutarate derived)
**glycerate**	Carbohydrate	Glycolysis
**glycerol**	Lipids	Glycerolipids
**glycerol 2-phosphate**	Lipids	Phospholipids
**glycerol 3-phosphate (G3P)**	Lipids	Phospholipids
**glycerophosphorylcholine (GPC)**	Lipids	Phospholipids
**glycine**	Amino acid	Serine family (phosphoglycerate derived)
**glycitein**	Secondary metabolism	Flavonoids
**glycitin**	Secondary metabolism	Flavonoids
**glycyltyrosine**	Peptide	Dipeptide
**guanine**	Nucleotide	Purine metabolism
**guanosine**	Nucleotide	Purine metabolism
**guanosine-2',3'-cyclic monophosphate**	Nucleotide	Purine metabolism
**gulono-1,4-lactone**	Cofactors, Prosthetic Groups, Electron Carriers	Ascorbate metabolism
**histidine**	Amino acid	Glutamate family (alpha-ketoglutarate derived)
**homoserine**	Amino acid	Aspartate family (OAA derived)
**inositol 1-phosphate (I1P)**	Carbohydrate	Inositol metabolism
**inositol 2-phosphate (I2P)**	Carbohydrate	Inositol metabolism
**isocitrate**	Carbohydrate	TCA cycle
**isoleucine**	Amino acid	Branched Chain Amino Acids (OAA derived)
**leucine**	Amino acid	Branched Chain Amino Acids (pyruvate derived)
**linoleate (18:2n6)**	Lipids	Free fatty acid
**linolenate [alpha or gamma; (18:3n3 or 6)]**	Lipids	Free fatty acid
**lysine**	Amino acid	Aspartate family (OAA derived)
**malate**	Carbohydrate	TCA cycle
**malonate (propanedioate)**	Lipids	Free fatty acid
**mannitol**	Carbohydrate	Sucrose, glucose, fructose metabolism
**methionine**	Amino acid	Aspartate family (OAA derived)
**methionine sulfoxide**	Amino acid	Aspartate family (OAA derived)
**methylphosphate**	Cofactors, Prosthetic Groups, Electron Carriers	Oxidative phosphorylation
**methylsuccinate**	Amino acid	Branched Chain Amino Acids (pyruvate derived)
**myo-inositol**	Carbohydrate	Inositol metabolism
**myo-inositol hexakisphosphate**	Carbohydrate	Inositol metabolism
**N6-acetyllysine**	Amino acid	Aspartate family (OAA derived)
**N-acetylornithine**	Amino acid	Glutamate family (alpha-ketoglutarate derived)
**naringenin**	Secondary metabolism	Flavonoids
**naringenin-7-O-glucoside**	Secondary metabolism	Flavonoids
**N-carbamoylaspartate**	Amino acid	Aspartate family (OAA derived)
**nicotinamide ribonucleotide (NMN)**	Cofactors, Prosthetic Groups, Electron Carriers	Nicotinate and nicotinamide metabolism
**nicotinamide riboside***	Cofactors, Prosthetic Groups, Electron Carriers	Nicotinate and nicotinamide metabolism
**nicotinate ribonucleoside***	Cofactors, Prosthetic Groups, Electron Carriers	Nicotinate and nicotinamide metabolism
**oleate (18:1n9)**	Lipids	Free fatty acid
**pantothenate**	Cofactors, Prosthetic Groups, Electron Carriers	CoA metabolism
**phenylalanine**	Amino acid	Aromatic amino acid metabolism (PEP derived)
**phosphate**	Cofactors, Prosthetic Groups, Electron Carriers	Oxidative phosphorylation
**p-hydroxybenzaldehyde**	Secondary metabolism	Benzenoids
**pinitol**	Carbohydrate	Inositol metabolism
**pipecolate**	Amino acid	Aspartate family (OAA derived)
**proline**	Amino acid	Glutamate family (alpha-ketoglutarate derived)
**putrescine**	Amino acid	Amines and polyamines
**pyridoxate**	Cofactors, Prosthetic Groups, Electron Carriers	Vitamin B metabolism (B6 or B12)
**pyroglutamine***	Amino acid	Glutamate family (alpha-ketoglutarate derived)
**pyroglutamylglutamine**	Peptide	Dipeptide
**pyruvate**	Carbohydrate	Glycolysis
**raffinose**	Carbohydrate	Sucrose, glucose, fructose metabolism
**ribitol**	Carbohydrate	Amino sugar and nucleotide sugar
**ribose**	Carbohydrate	Amino sugar and nucleotide sugar
**scyllo-inositol**	Carbohydrate	Inositol metabolism
**serine**	Amino acid	Serine family (phosphoglycerate derived)
**sorbitol**	Carbohydrate	Sucrose, glucose, fructose metabolism
**spermidine**	Amino acid	Amines and polyamines
**stachyose**	Carbohydrate	Sucrose, glucose, fructose metabolism
**stearamide**	Lipids	Fatty acid amide
**stigmasterol**	Secondary metabolism	Terpenoids
**succinate**	Carbohydrate	TCA cycle
**sucrose**	Carbohydrate	Sucrose, glucose, fructose metabolism
**syringic acid**	Secondary metabolism	Flavonoids
**threitol**	Carbohydrate	Amino sugar and nucleotide sugar
**threonate**	Cofactors, Prosthetic Groups, Electron Carriers	Ascorbate metabolism
**threonine**	Amino acid	Aspartate family (OAA derived)
**trans-4-hydroxyproline**	Amino acid	Glutamate family (alpha-ketoglutarate derived)
**trigonelline (N'-methylnicotinate)**	Cofactors, Prosthetic Groups, Electron Carriers	Nicotinate and nicotinamide metabolism
**tryptophan**	Amino acid	Aromatic amino acid metabolism (PEP derived)
**tyrosine**	Amino acid	Aromatic amino acid metabolism (PEP derived)
**uridine**	Nucleotide	Pyrimidine metabolism
**uridine-2',3'-cyclic monophosphate**	Nucleotide	Pyrimidine metabolism
**valine**	Amino acid	Branched Chain Amino Acids (pyruvate derived)
**valylglutamate**	Peptide	Dipeptide
**vanillate**	Secondary metabolism	Benzenoids
**verbascose**	Carbohydrate	Sucrose, glucose, fructose metabolism
**xanthosine**	Nucleotide	Purine metabolism
**xylitol**	Carbohydrate	Amino sugar and nucleotide sugar
**xylonate**	Carbohydrate	Amino sugar and nucleotide sugar

Amino acid: Serine Family (PGA derived)

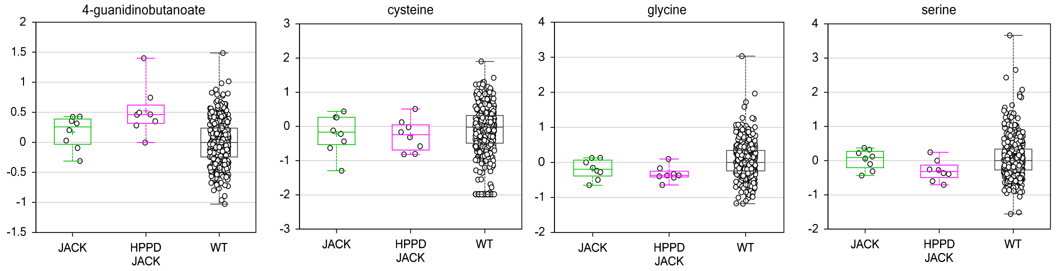

Amino acid: Aromatic (PEP derived)

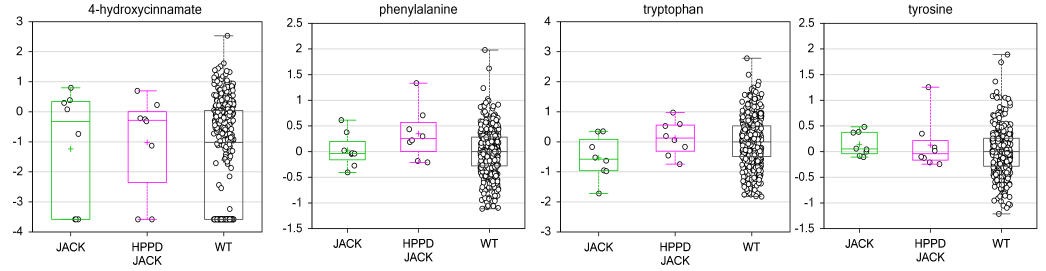

Amino acid: Aspartate Family (OAA derived)

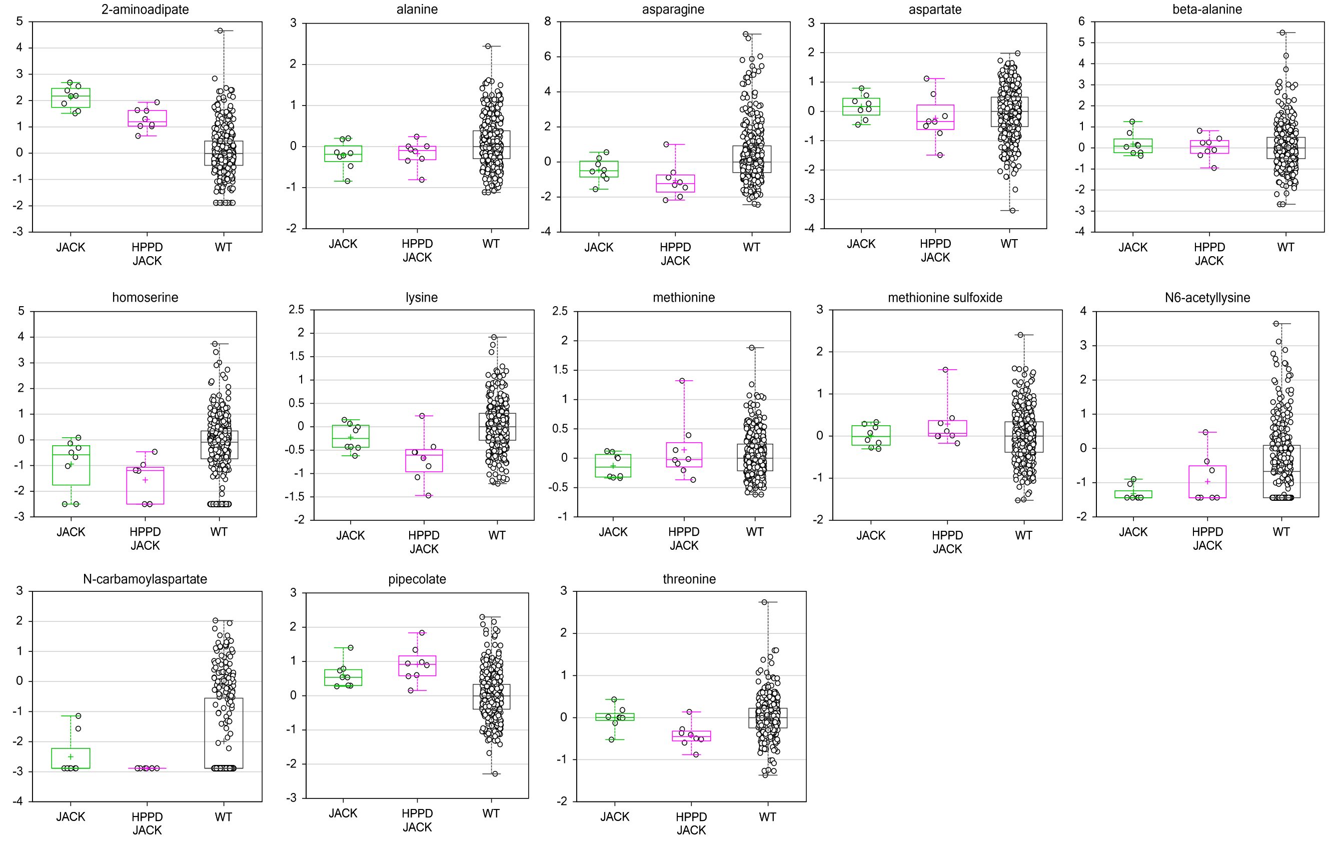

Amino acid: Glutamate Family (αKG derived)

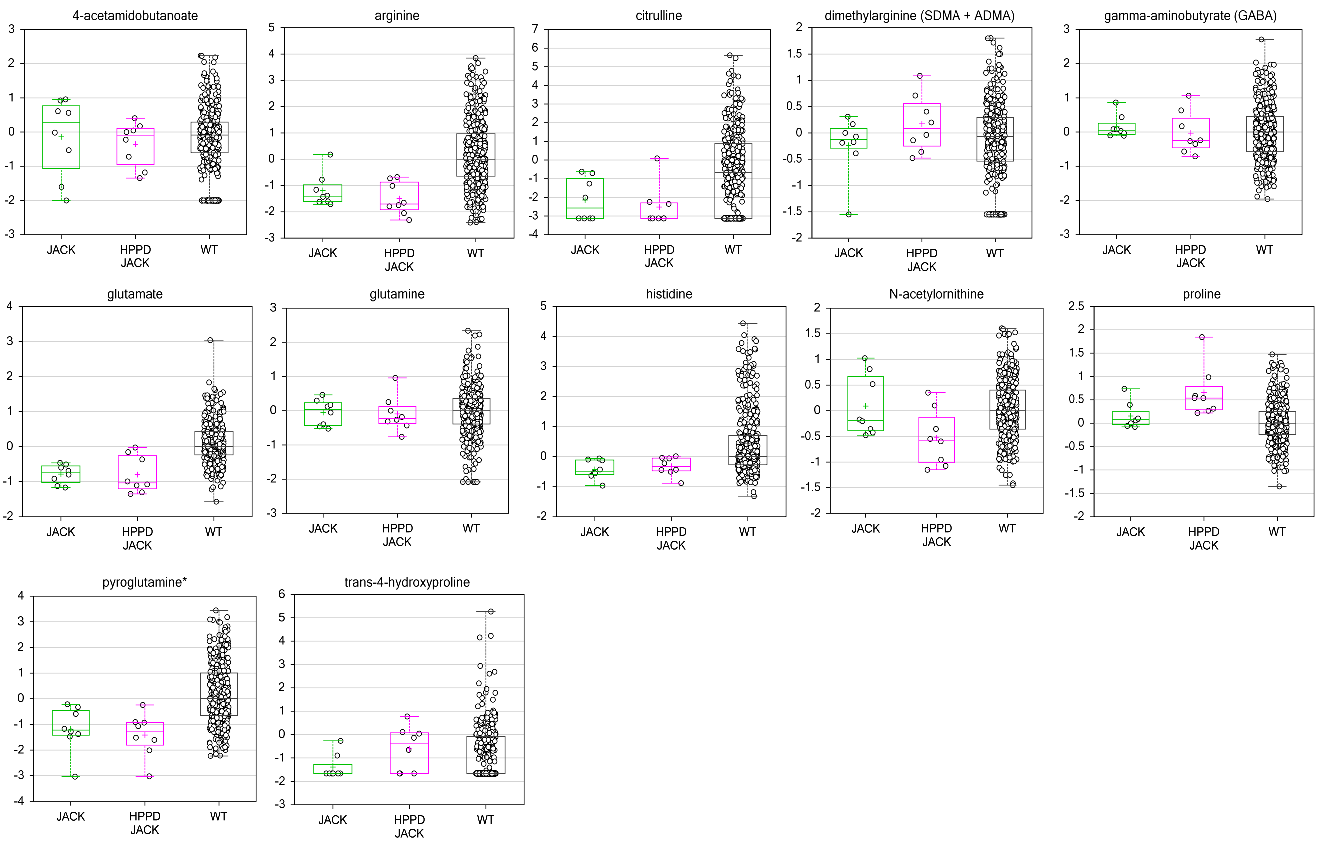

Amino acid: BCAA (OAA derived)

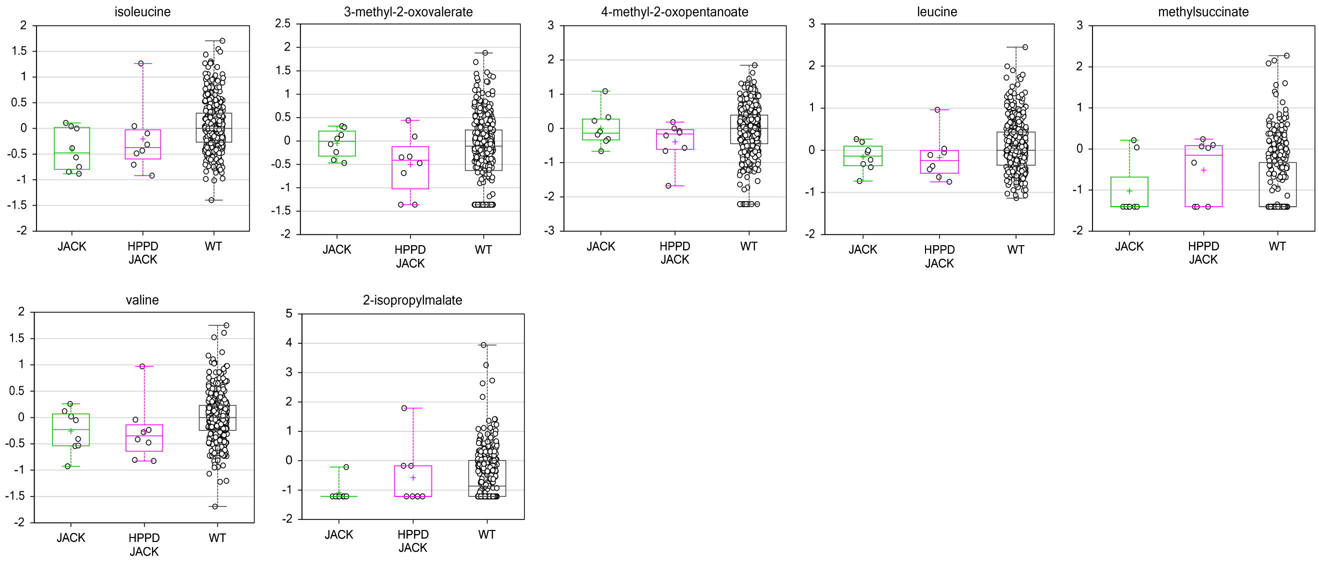

Amino acid: Amines and polyamines

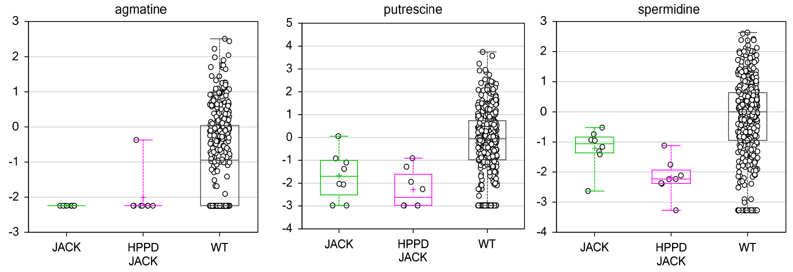

Amino acid: Glutathione metabolism

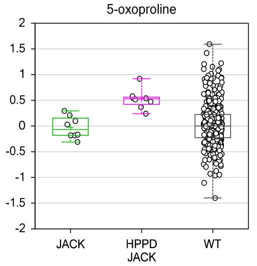

Carbohydrate: Glycolysis

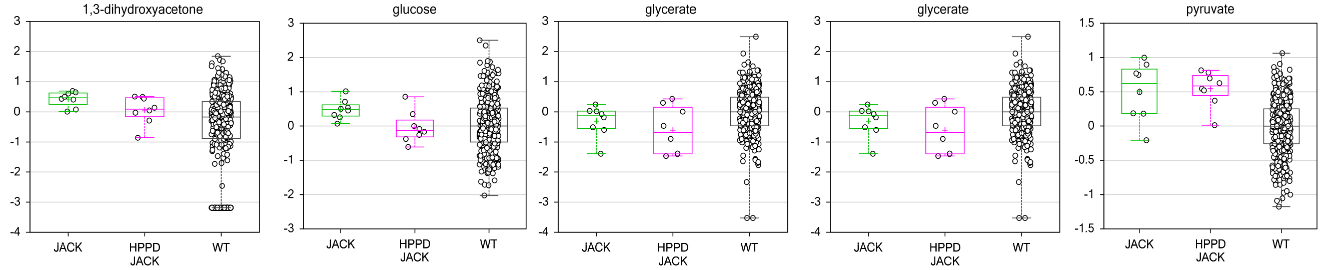

Carbohydrate: TCA Cycle

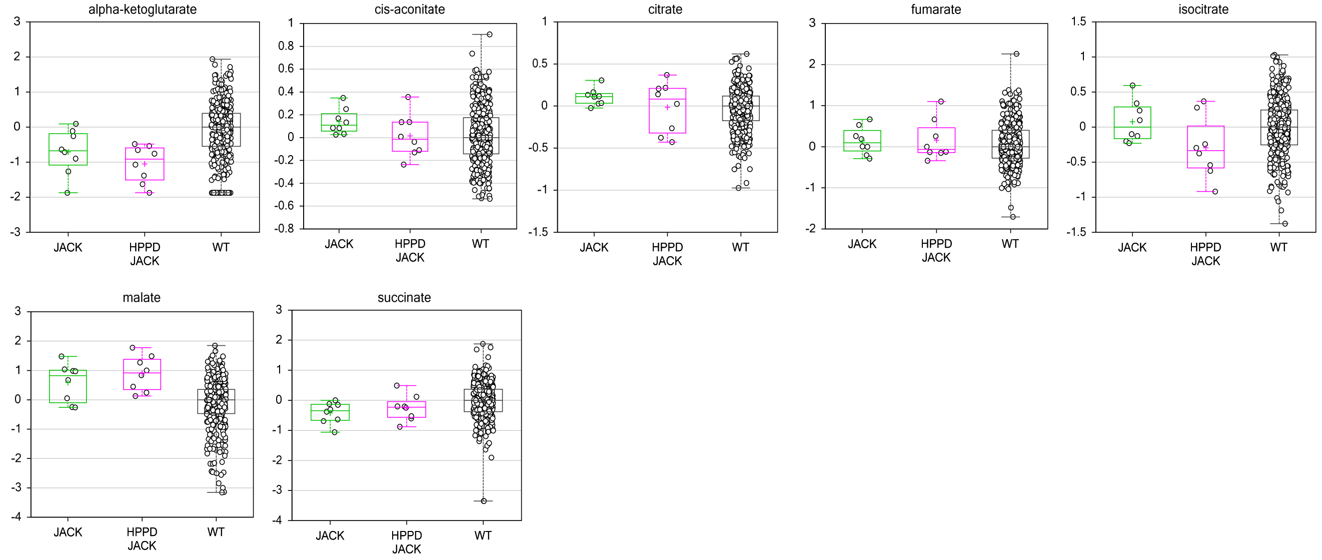

Carbohydrate: Amino sugars; nucleotides sugars

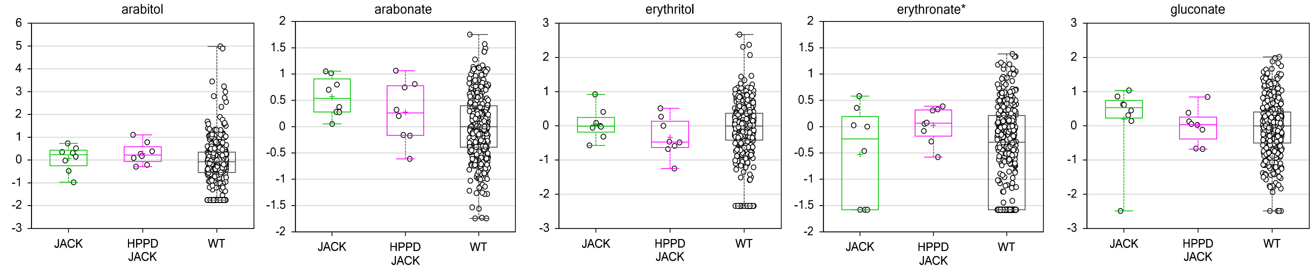

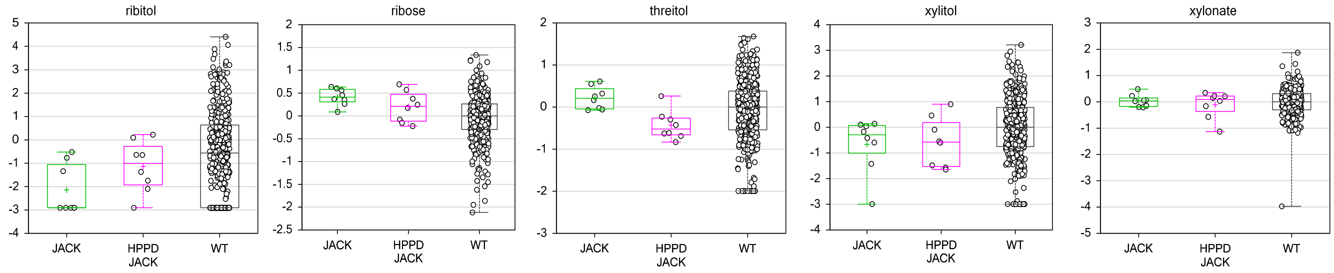

Carbohydrate: Inositol metabolism

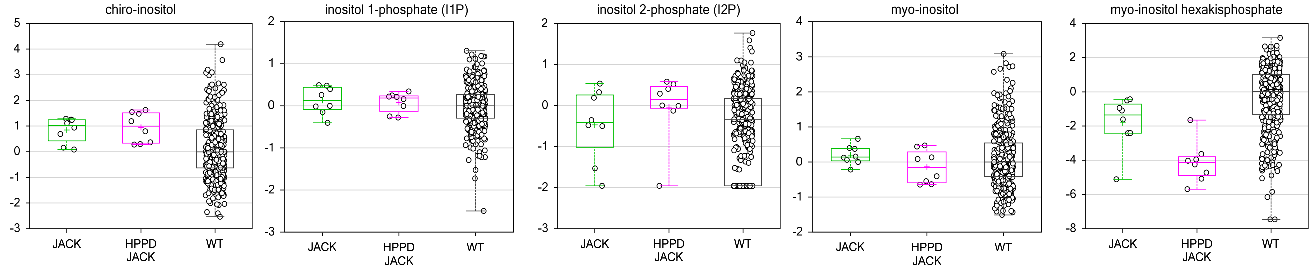

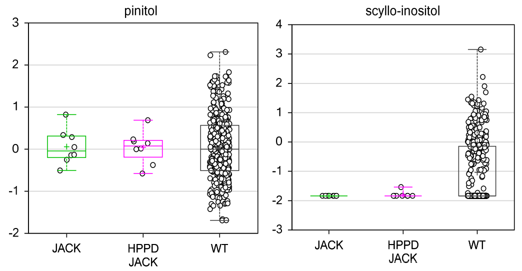

Carbohydrate: C5 branched dibasic acid metabolism

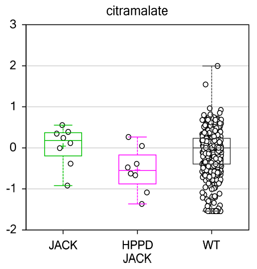

Carbohydrate: Sucrose, glucose, fructose metabolism

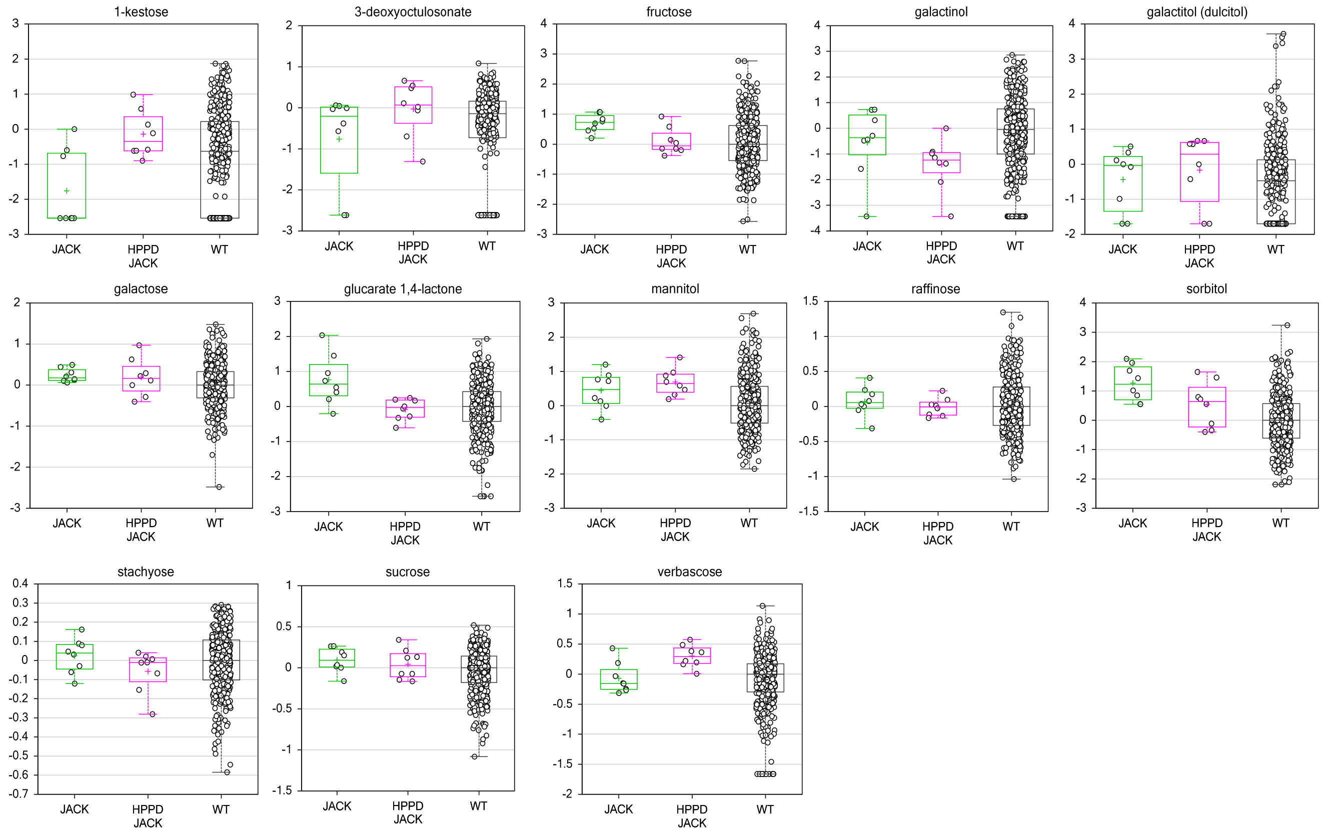

Lipids: Free fatty acids

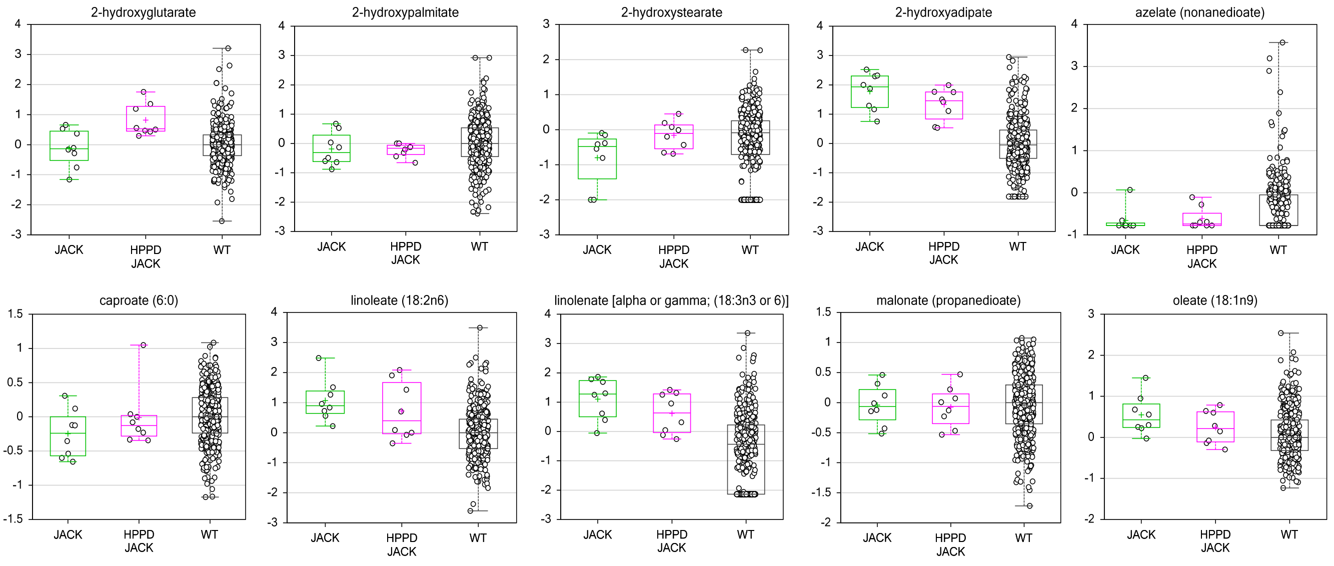

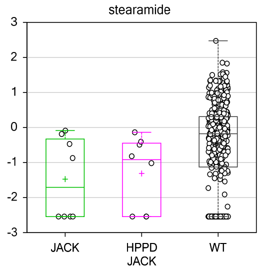

Lipids: Oxylipins

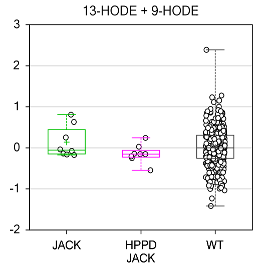

Lipids: Glycerolipids

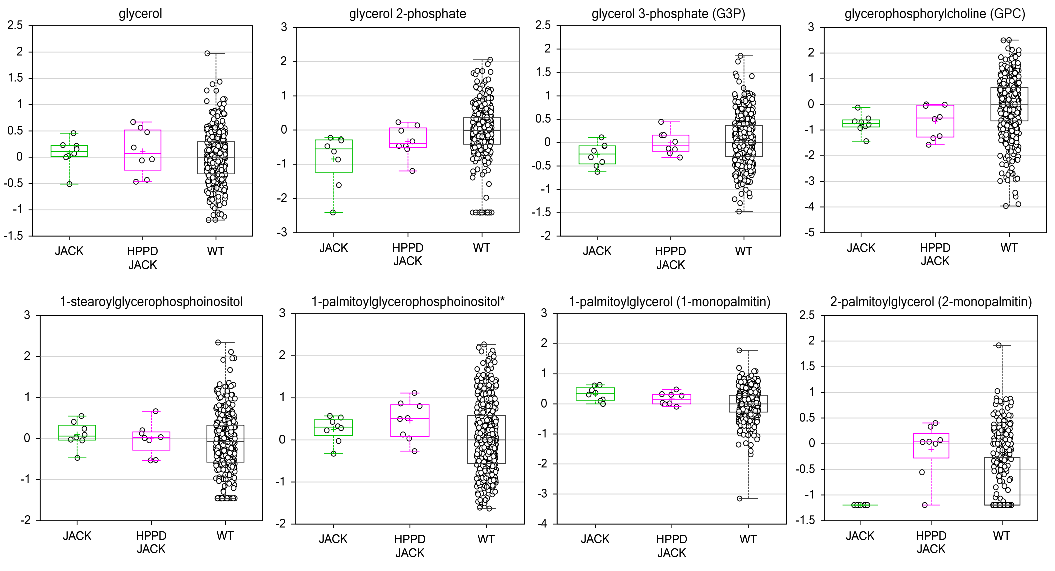

Lipids: Choline metabolism

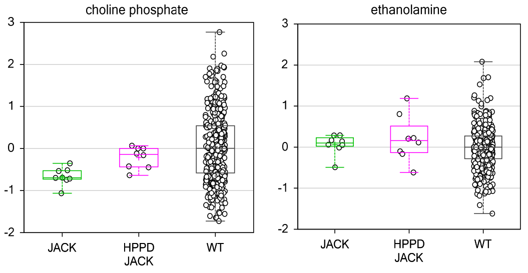

Lipids: Sterols

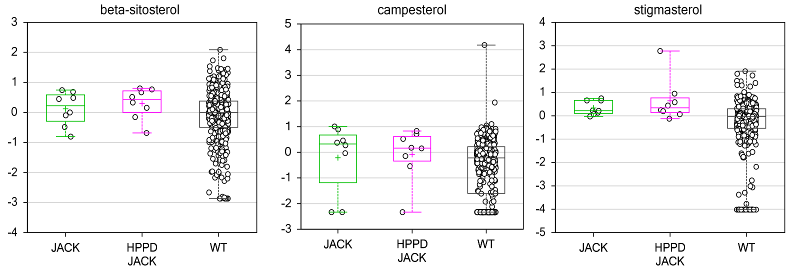

Cofactors, Prosthetic Groups, Electron carriers: Nicotinate and nicotinamide metabolism

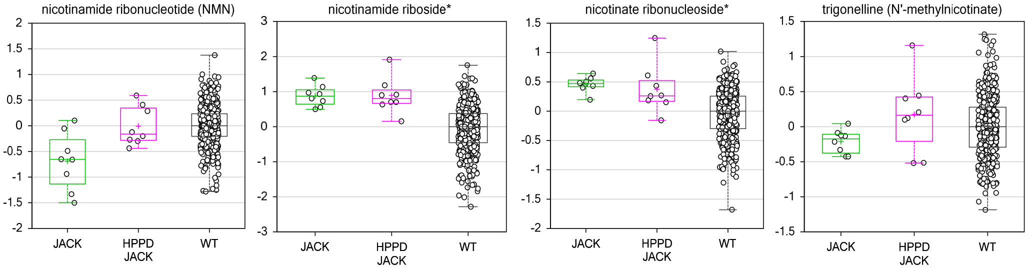

Cofactors, Prosthetic Groups, Electron carriers: CoA metabolism

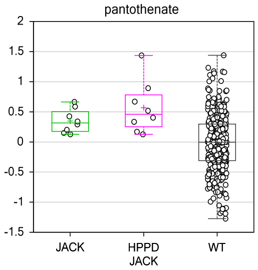

Cofactors, Prosthetic Groups, Electron carriers: Oxidative phosphorylation

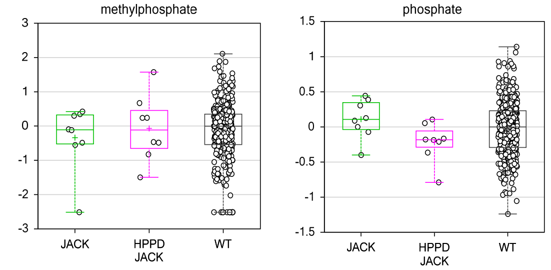

Cofactors, Prosthetic Groups, Electron carriers: Ascorbate metabolism

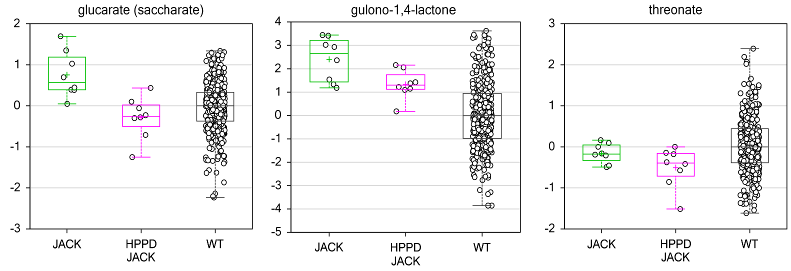

Cofactors, Prosthetic Groups, Electron carriers: Tocopherol metabolism

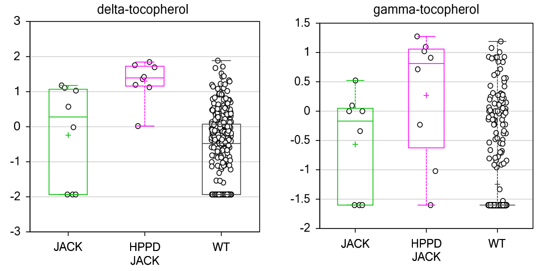

Cofactors, Prosthetic Groups, Electron carriers: Vitamin B metabolism

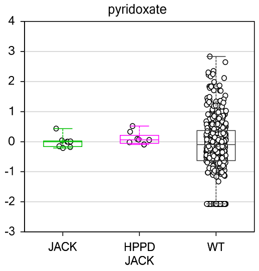

Nucleotides: Purine metabolism

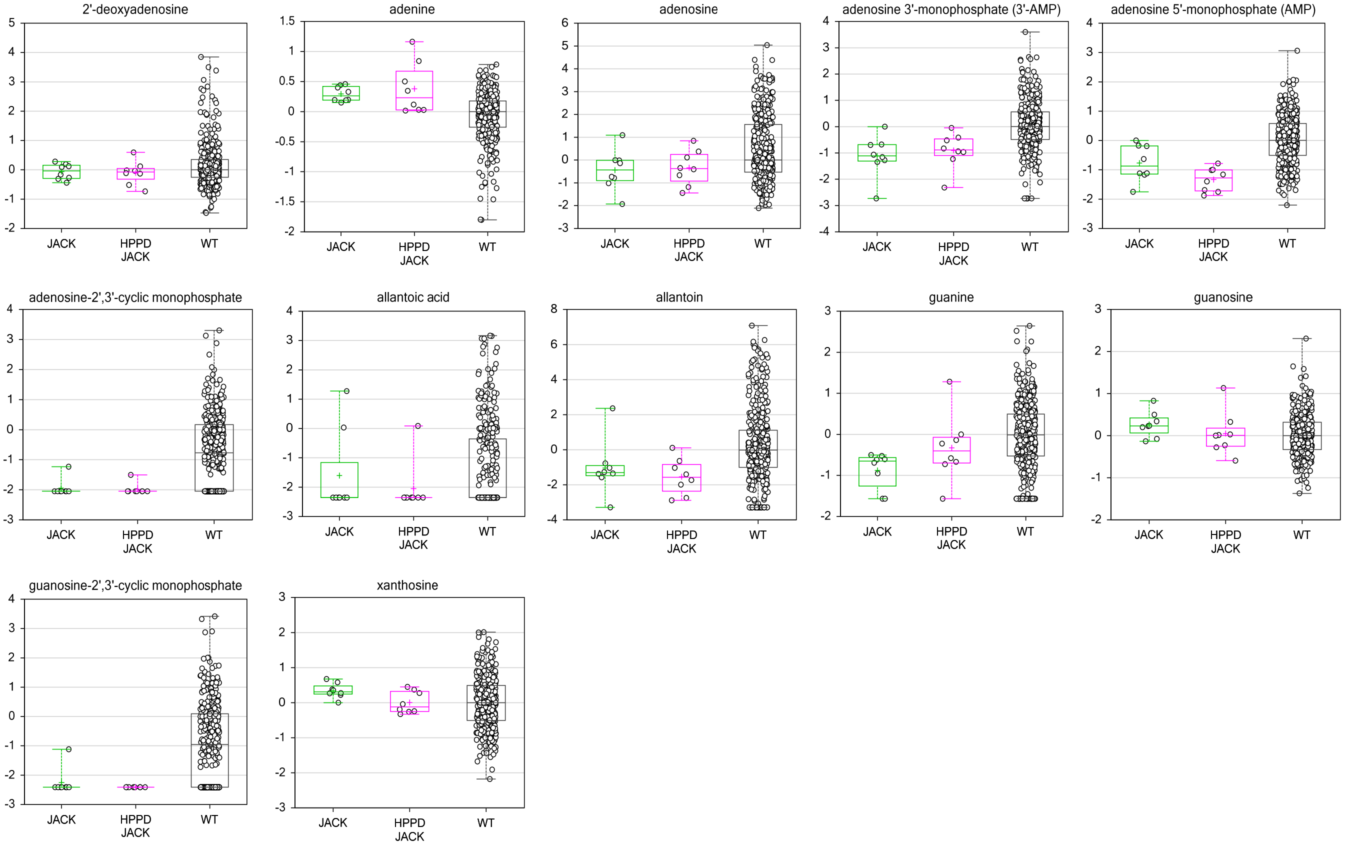

Nucleotides: Pyrimidine metabolism

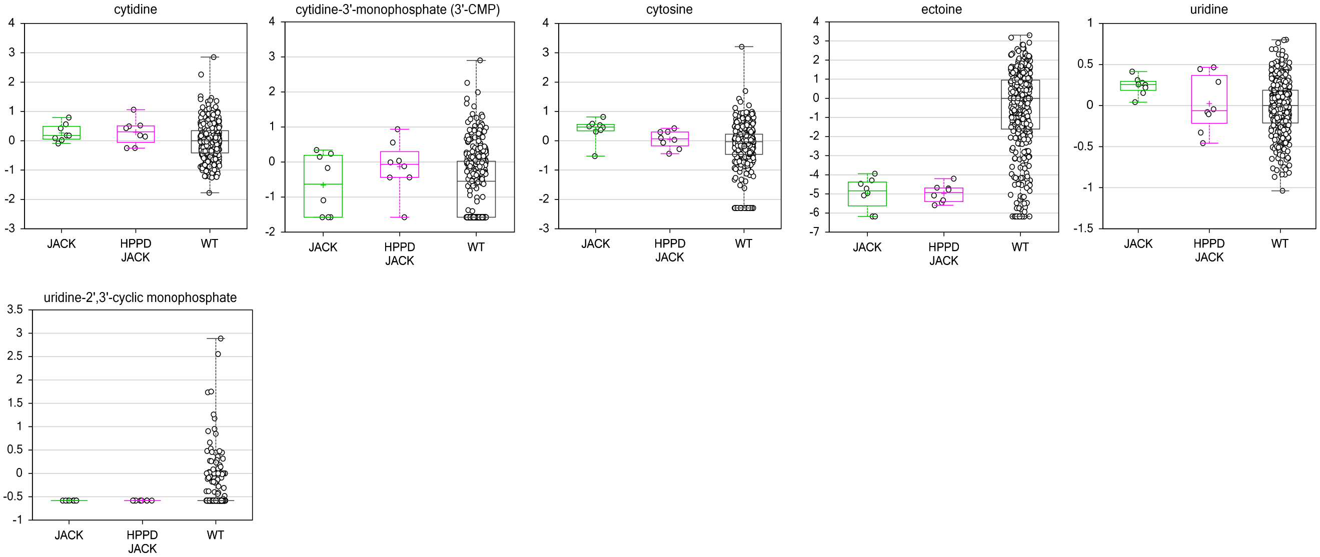

Peptides: *Alpha*-dipeptides

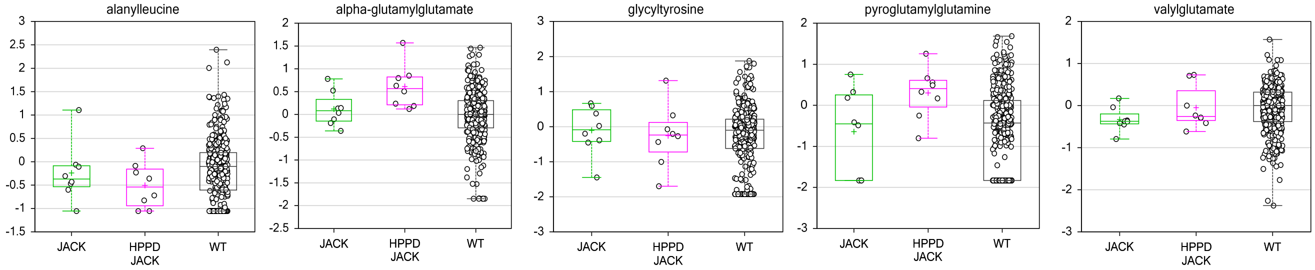

Peptides: *Gamma*-glutamyl amino acids

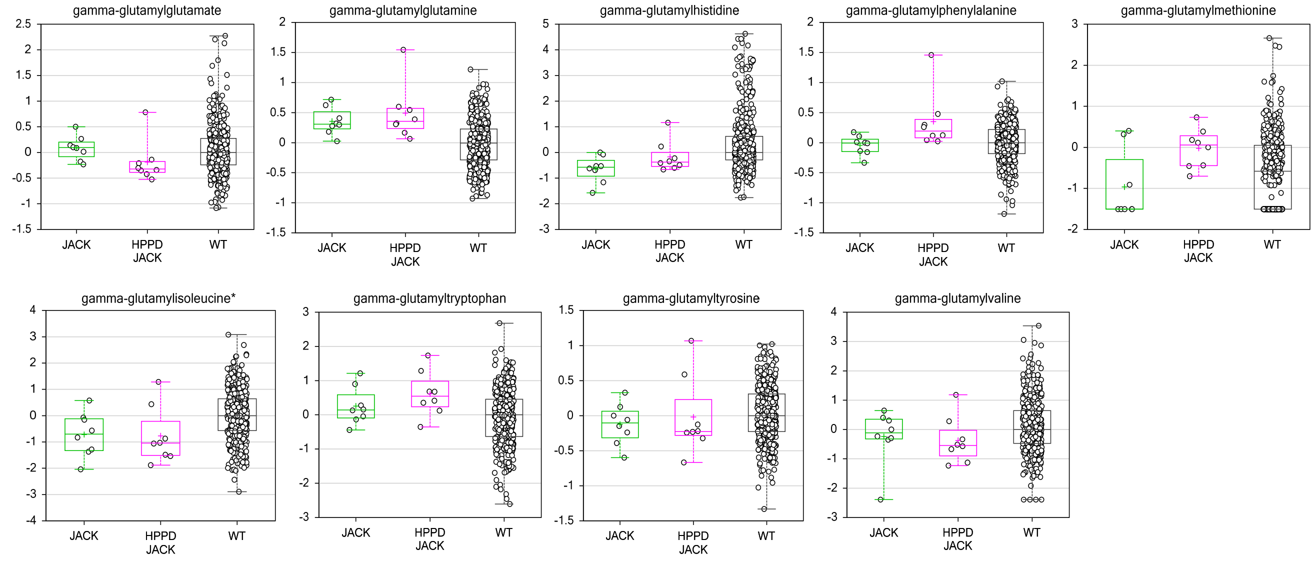

Secondary metabolism: Benzenoids

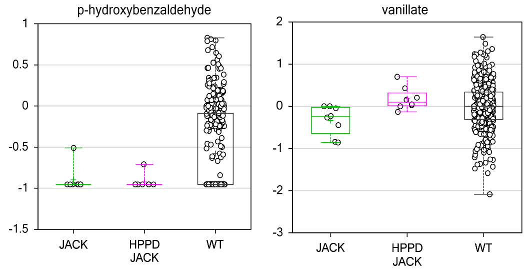

Secondary metabolism: Fatty acid or sugar derivatives

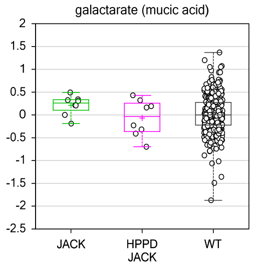

Secondary metabolism: Flavonoids

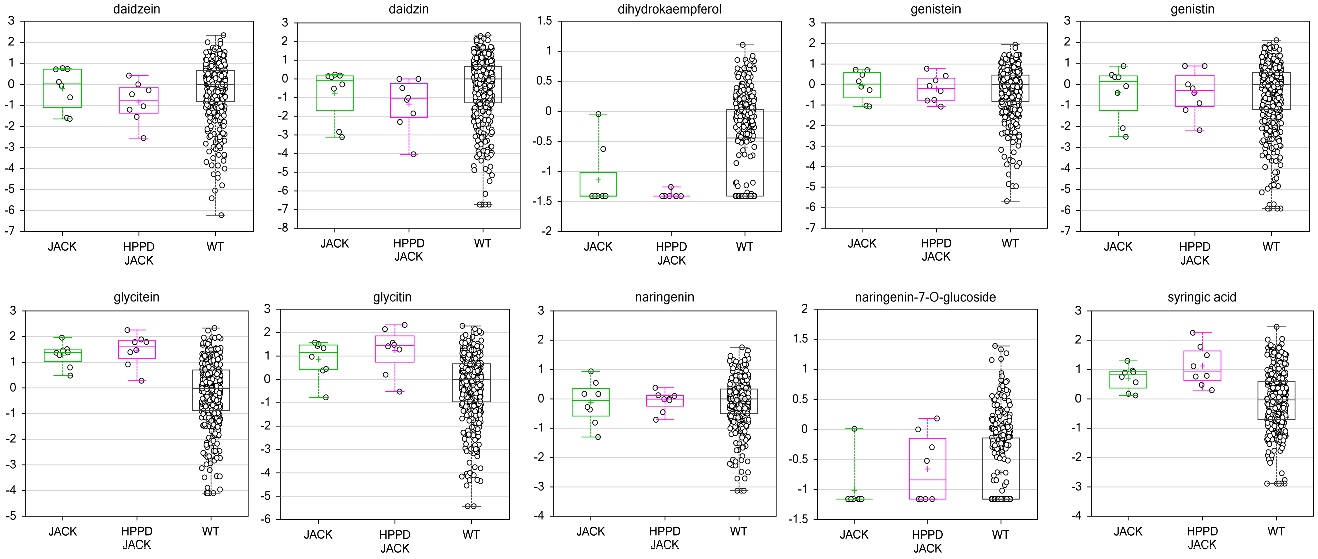

Supplement: Supplementary Information — supplemental material [file srep03082-s1.pdf]
